# NLRP12 is an innate immune checkpoint for repressing IFN signatures and attenuating lupus nephritis progression

**DOI:** 10.1172/JCI157272

**Published:** 2023-02-01

**Authors:** Yen-Po Tsao, Fang-Yu Tseng, Chih-Wei Chao, Ming-Han Chen, Yi-Chen Yeh, Babamale Olarewaju Abdulkareem, Se-Yi Chen, Wen-Ting Chuang, Pei-Ching Chang, I-Chun Chen, Pin-Hsuan Wang, Chien-Sheng Wu, Chang-Youh Tsai, Szu-Ting Chen

**Affiliations:** 1 Institute of Clinical Medicine, National Yang Ming Chiao Tung University, Hsinchu, Taiwan.; 2 Division of Allergy, Immunology and Rheumatology, Department of Medicine, and; 3 Division of Holistic and Multidisciplinary Medicine, Department of Medicine, Taipei Veterans General Hospital, Taipei, Taiwan.; 4 Program in Molecular Medicine, National Yang Ming Chiao Tung University and Academia Sinica, Taipei, Taiwan.; 5 Department of Pathology and Laboratory Medicine, Taipei Veterans General Hospital, Taipei, Taiwan.; 6 Taiwan International Graduate Program in Molecular Medicine, National Yang Ming Chiao Tung University and Academia Sinica, Taipei, Taiwan.; 7 Department of Neurosurgery, and; 8 School of Medicine, Chung-Shan Medical University, Taichung, Taiwan.; 9 Institute of Microbiology and Immunology, National Yang Ming Chiao Tung University, Hsinchu, Taiwan.; 10 Department of Internal Medicine, Far Eastern Memorial Hospital, New Taipei City, Taiwan.; 11 Cancer Progression Research Center, National Yang Ming Chiao Tung University, Hsinchu, Taiwan.

**Keywords:** Autoimmunity, Inflammation, Cytokines, Innate immunity, Lupus

## Abstract

Signaling driven by nucleic acid sensors participates in interferonopathy-mediated autoimmune diseases. NLRP12, a pyrin-containing NLR protein, is a negative regulator of innate immune activation and type I interferon (IFN-I) production. Peripheral blood mononuclear cells (PBMCs) derived from systemic lupus erythematosus (SLE) patients expressed lower levels of *NLRP12*, with an inverse correlation with *IFNA* expression and high disease activity. *NLRP12* expression was transcriptionally suppressed by runt-related transcription factor 1–dependent (RUNX1-dependent) epigenetic regulation under IFN-I treatment, which enhanced a negative feedback loop between low NLRP12 expression and IFN-I production. Reduced NLRP12 protein levels in SLE monocytes was linked to spontaneous activation of innate immune signaling and hyperresponsiveness to nucleic acid stimulations. Pristane-treated Nlrp12^–/–^ mice exhibited augmented inflammation and immune responses; and substantial lymphoid hypertrophy was characterized in NLRP12-deficient lupus-prone mice. NLRP12 deficiency mediated the increase of autoantibody production, intensive glomerular IgG deposition, monocyte recruitment, and the deterioration of kidney function. These were bound in an IFN-I signature–dependent manner in the mouse models. Collectively, we reveal a remarkable link between low *NLRP12* expression and lupus progression, which suggests the impact of NLRP12 on homeostasis and immune resilience.

## Graphical abstract

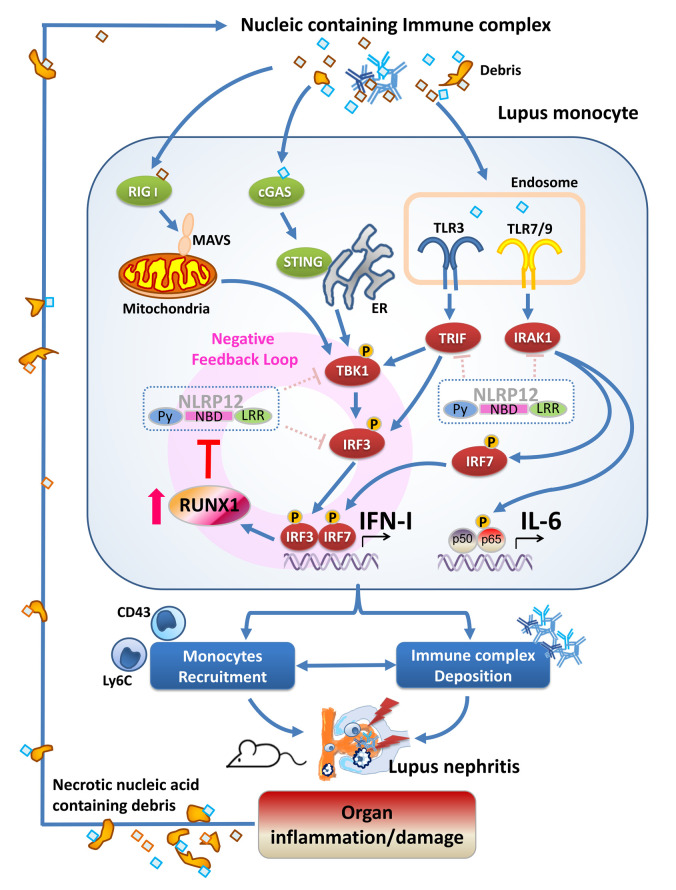

## Introduction

Systemic lupus erythematosus (SLE) is a multifactorial autoimmune disease with unknown etiology involving immune intolerance of endogenous nuclear materials, increased levels of autoreactive B cells, and chronic inflammation, leading to systemic autoimmunity and organ damage. The differential efficacy of rituximab in moderately to severely active SLE patients confirms the existence of a B cell–independent mechanism with functions beyond the adaptive immune response (1). In lupus, nucleic acid–containing complexes in the serum were observed to activate the nucleic acid–recognizing TLRs, i.e., TLR7 and TLR9, which are chiefly expressed by immune cells (2, 3). TLR7 is an endosomal sensor of foreign RNA that triggers the production of proinflammatory cytokines and type I interferon (IFN-I). The self-nucleic acids in SLE patients are associated with the generation of autoantibodies to form immune complexes (ICs) that aberrantly activate TLR7 signaling in the absence of foreign RNA (4). Mouse studies unveiled the critical roles of TLR7 in the pathogenesis of SLE, including that (a) its constitutive activation drives systemic autoimmunity (5); (b) deficiency of TLR7 reduces the generation of autoantibodies to RNA-containing antigens and the development of glomerulonephritis (GN) in pristane-induced lupus nephritis (LN) (6); and (c) lupus-prone mice with TLR7 deficiency (*Tlr7^–/–^lpr/lpr*) exhibit decreased lymphocyte activation and IgG levels in serum (7). Similarly, DNA-sensing pathways are also crucial for SLE onset (8). Cyclic GMP–AMP synthase (cGAS) and stimulator of interferon genes (STING) are involved in DNA recognition and drive IFN-I production and the expression of IFN-stimulated genes (ISGs). Kato et al. demonstrated that both elevated dsDNA and apoptosis-derived membrane vesicle levels in serum from SLE patients induced high ISG-inducing activity, which was diminished in cGAS-knockout or STING-knockout reporter cells (3). Additionally, cGAS activation by self-DNA has been indicated in *Sting^–/–^DNaseII^–/–^* and *cGAS^–/–^DNaseII^–/–^* models with reduced lupus-like manifestations (8, 9). Accordingly, the DNA component of SLE-ICs induces overproduction of IFN-I and chemokines in plasmacytoid DCs (pDCs) (10), and defective clearance of self-derived nucleic acids can cause systemic inflammation and severe IFN-associated autoimmunity in humans and mice (11). All of these factors strengthen the roles of nucleic acid sensors and signaling in IFN-I production in the promotion of SLE. Increased expression of IFN-I–related genes and ISGs was observed in the peripheral blood mononuclear cells (PBMCs) of SLE patients using gene expression profiling, particularly in the majority of pediatric (>95%) and 50%–70% of adult SLE patients (12). IFN-I has an impact on both host innate and adaptive immune responses, as it can disrupt the self-tolerance associated with DC differentiation and antigen presentation, leading to the unnecessary activation of T and B cells (13). In addition, IFN-I–primed neutrophils exhibited robust neutrophil extracellular trap (NET) formation in response to IC stimulation; NETs containing genomic DNA induce the production of IFN-I in pDCs, and nuclear proteins serve as a source of self-antigens (14). Clinically, IFN-I signatures are associated with the production of anti-ribonucleoprotein (anti-RNP) and anti-dsDNA Abs in SLE patients (15). Therefore, tight regulation of IFN production and signaling cascades is key to maintaining homeostasis and preventing excessive immune responses. NLR family pyrin domain–containing 12 (NLRP12), which is primarily expressed by the myeloid cell lineage, was recently identified as an essential negative regulator of innate immune pathways, including TLR- and non-TLR–derived canonical and noncanonical NF-κB activation (16). Studies of NLRP12 revealed its notable regulatory mechanisms, necessity in host defense, role in disease progression, and involvement in the restoration of physical homeostasis (17). During viral infection, NLRP12-mediated inhibition is relieved due to its reduced expression, which activates retinoic acid–inducible gene I–mediated (RIG-I–mediated) signaling. In the absence of NLRP12, the increased levels of phosphorylated NF-κB, TANK-binding kinase 1 (TBK1), and IFN regulatory factor 3 (IRF-3) associated with the corresponding host antiviral response are activated and/or accelerated (18). The elevated immune signaling observed in *Nlrp12^–/–^* mice contributes to the greater immune activation and IFN production observed in response to nucleic acid stimulation that imitates the impetus of interferonopathy-based autoimmunity. Previous studies have associated the uncontrolled activation of nucleic acid sensors and signaling with autoimmune diseases resulting from elevated IFN levels and their biological consequences (9). Therefore, we proposed that a low expression level of *NLRP12* in immune cells was a regulatory target of nucleic acid sensors and signaling during the production of proinflammatory cytokines and IFN-I in inflammatory diseases. Here, we report that PBMCs from SLE patients exhibited lower levels of *NLRP12* mRNA than those from healthy donors. This finding is inversely correlated with *IFNA* transcription. The consistently low *NLRP12* expression observed in SLE PBMCs and high expression levels of IFN signature genes in SLE patients indicate a negative feedback loop that regulates homeostatic conditions. Animal studies confirmed the finding that dysregulated/low *NLRP12* expression causes an uncontrolled inflammatory reaction and IFN-I production, which not only accelerates disease progression, but also exacerbates the pathogenesis of lupus in animals.

## Results

### PBMCs from SLE patients exhibit low NLRP12 expression.

**Figure 1:**
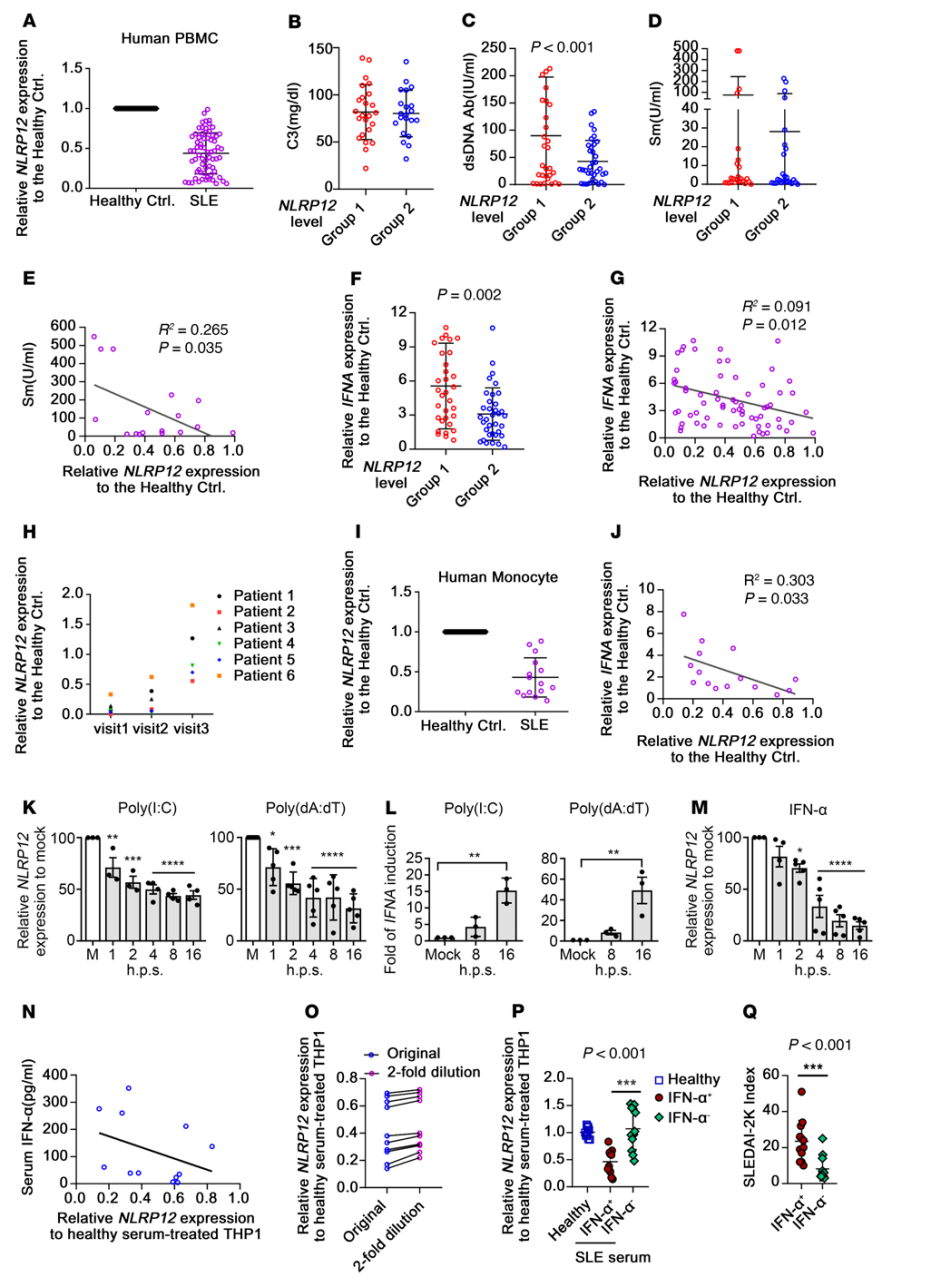
PBMCs from SLE patients exhibit low *NLRP12* expression. (**A**) *NLRP12* expression in SLE PBMCs relative to healthy controls was determined by quantitative reverse-transcriptase PCR (RT-qPCR). Relative *NLRP12* expression was analyzed by the ΔΔCt = (Δ*C_SLE*–CtNorm_(health)_) and 2^(–ΔΔCt)^ algorithm, and *NLRP12* expression level in healthy controls was set as 1. Levels of (**B**) complement C3, (**C**) anti-dsDNA, and (**D**) anti-Sm Abs and corresponding *NLRP12* expression were grouped and are shown. (**E**) Regression of *NLRP12* expression and anti-Sm Abs. (**F**) Relative *IFNA* expression and corresponding *NLRP12* expression were grouped and are shown. (**G**) Regression of *NLRP12* and *IFNA* expression in SLE PBMCs. (**H**) Relative *NLRP12* expression in PBMCs from SLE patients at visit follow-up. (**I**) *NLRP12* expression in SLE monocytes relative to healthy monocytes. (**J**) Regression assay of *NLRP12* and *IFNA* expression in SLE monocytes. (**K** and **L**) THP-1 cells were transfected with poly(I:C) and poly(dA:dT). *NLRP12* and *IFNA* expression were measured. (**M**) *NLRP12* expression of the IFN-α2–treated THP-1 cells. (**N**) Regression of *NLRP12* expression of serum-treated THP-1 and levels of IFN-α in corresponding serum. (**O**) *NLRP12* expression of serum-treated or 2-fold diluted serum-treated THP-1 cells. (**P**) THP-1 cells were treated with healthy (*n* = 10) or SLE (*n* = 24) sera. (**Q**) Sera from SLE patients (*n* = 24) with and without detectable IFN-α were grouped, and corresponding SLEDAI-2K was recorded. (**B**, **C**, and **F**) Two-tailed Student’s *t* test; (**D**, **P**, and **Q**) Mann-Whitney *U* test; (**E**, **G**, **J**, and **N**) Spearman’s correlation; (**K**–**M**) 1-way ANOVA test (multiple samples with mock control). Data are represented as mean ± SEM. *P < 0.05; **P < 0.01; ***P < 0.001; ****P < 0.0001.

To measure the expression levels of *NLRP12* in SLE patients, PBMCs from 34 healthy controls and 68 SLE patients who satisfied the 2012 Systemic Lupus International Collaborating Clinics (SLICC) classification criteria (19) were collected (Supplemental Table 1; supplemental material available online with this article; https://doi.org/10.1172/JCI157272DS1). Lupus patients with active bacterial or viral infection and active malignancy affecting the IFN signature were excluded from this study. Here, we showed that the relative expression of *NLRP12* in SLE patient–derived PBMCs was significantly lower than that in healthy PBMCs (control PBMCs) (Figure 1A). Among the PBMCs obtained from 68 patients, the values of the relative *NLRP12* expression displayed a normal distribution, and the median value was 0.43. We used 0.43 as a cutoff to divide SLE patients into groups 1 (<0.43) and 2 (>0.43) based on their relative *NLRP12* expression levels. We further evaluated serological markers for lupus, including complement 3 (C3), anti-dsDNA and anti-Smith (anti-Sm) Abs. There were no differences in C3 levels between groups 1 and 2 (Figure 1B), but a significantly higher level of anti-dsDNA Abs was observed in group 1 (Figure 1C). Anti-Sm Ab is a confirmed marker for SLE diagnosis, and positivity for this marker is observed in 20%–30% of SLE patients (20). Among 68 patients enrolled, there were 17 patients with an anti-Sm Ab level above 7 U/mL, and there were no significant differences in the anti-Sm Ab levels between groups 1 and 2 (Figure 1D). Therefore, we conducted regression analysis using those 17 samples, and a significant inverse correlation (Figure 1E) was observed. The relative *IFNA* expression in the SLE patient–derived PBMCs compared with the control PBMCs was measured. SLE patients in group 1 had higher *IFNA* expression levels than those in group 2 (Figure 1F), and linear regression analysis with *NLRP12* expression showed a significant inverse correlation in SLE patient–derived PBMCs (Figure 1G). Among 68 SLE patients enrolled, 6 patients with unstable disease activity who underwent treatments, including mycophenolate mofetil, steroids, and azathioprine, were followed up at their posttreatment hospital visits. Importantly, the levels of *NLRP12* gradually increased, with improved lupus disease activity after subsequent visits (Figure 1H). This observation revealed that *NLRP12* expression in PBMCs might act as a measurable marker for treatment response. *NLRP12* and *IFNA* expression levels were measured in monocytes isolated from SLE patients and healthy donors. SLE patient–derived monocytes exhibited lower *NLRP12* expression than control monocytes (Figure 1I), and *NLRP12* and *IFNA* expression levels exhibited a significant inverse correlation (Figure 1J). In summary, low *NLRP12* expression correlates with high *IFNA* expression in SLE patient–derived PBMCs and higher Ab titers to dsDNA and Sm, which are positively correlated with the disease activity of SLE and the potential deterioration of kidney function. We investigated the possible cause of reduced *NLRP12* expression in patient-derived PBMCs with the speculation that genomic materials might be involved (18). THP-1, a human monocytic cell line, was used to measure the change in *NLRP12* expression, and infection with the RNA virus (*Vesicular stomatitis* virus [VSV]) and the DNA virus (*Herpes simplex virus-1*) downregulated *NLRP12* expression (Supplemental Figure 1A). We attributed the reduced *NLRP12* expression to the nucleic acid components derived from viral infection; thus, we stimulated cells with a synthetic analog of dsRNA (poly[I:C]) or transfected cells with synthetic analogs of dsDNA sequences, such as poly(dA:dT) and poly(dG:dC). All these analogs caused a reduction in *NLRP12* levels and induced the expression of *IFNA* in THP-1 cells (Figure 1, K and L, and Supplemental Figure 1A). We also investigated the impact of TLR agonists TLR7/8 (CL097) and TLR9 (ODN2216), but none of them affected either *NLRP12* or *IFNA* expression in THP-1 cells (Supplemental Figure 1, B and C). These findings raised the possibility of the existence of a negative feedback loop involving *NLRP12* and *IFNA* expression. To confirm this, THP-1 cells were treated with IFN-α2, and *NLRP12* expression was significantly reduced at 4 hours until 16 hours (Figure 1M). We then evaluated whether serum derived from patients could reduce *NLRP12* levels in cells because serum derived from SLE patients with elevated IFN-I bioactivity can induce the expression of ISGs through the cGAS/STING pathway (3). Eighty-five serum samples were collected, and the levels of IFN-α were measured by an ELISA kit with a detection sensitivity ranging from 250 to 6.25 pg/mL. There were 12 samples with detectable IFN-α levels. Sera from healthy (*n* = 12) and SLE patients were used to treat THP-1 cells, followed by measuring relative *NLRP12* expression. The IFN-α–containing sera were able to reduce *NLRP12* expression in THP-1 cells even though the levels of suppression did not exactly correlate with the serum IFN-α levels (Figure 1N). *NLRP12* expression in THP-1 cells treated with a half-dose of patient-derived sera was slightly higher than that in cells treated with the original concentration (Figure 1O), suggesting that the dose effect of serum IFN-α levels might be involved in determining *NLRP12* expression in SLE patients (IFN-α level >20 pg/ml). To confirm the effect of serum IFN-α in reducing *NLRP12* expression, THP-1 cells were treated with sera from another 12 SLE patients in whom the IFN-α levels were undetectable (Supplemental Table 2), and those sera were unable to reduce *NLRP12* expression (Figure 1P). We measured IFN-I bioactivity in sera by treating healthy CD14^+^ monocytes with patient-derived serum and measuring IFN-α production. None of the SLE patient–derived serum induced CD14^+^ monocytes to secrete IFN-α, but their PBMCs still exhibited lower *NLRP12* levels than the control PBMCs, as shown in Figure 1A. This finding suggested the existence of yet-unknown factors in addition to IFN-α and nucleic acids that are required to cause the downregulation of *NLRP12* expression. The disease activity index (Systemic Lupus Erythematosus Disease Activity Index 2000, SLEDAI-2K) (21) is a modified score that comprises composite elements resulting from assessments of damages in different organs and clinical manifestations in lupus patients. The SLEDAI-2K was significantly higher in patients with detectable IFN-α levels in serum than in those with undetectable IFN-α levels in serum, and the levels of SLEDAI-2K were associated with the capacity of patient serum to downregulate *NLRP12* expression (Figure 1Q).

### The NLRP12 promoter contains RUNX1-binding sites.

**Figure 2:**
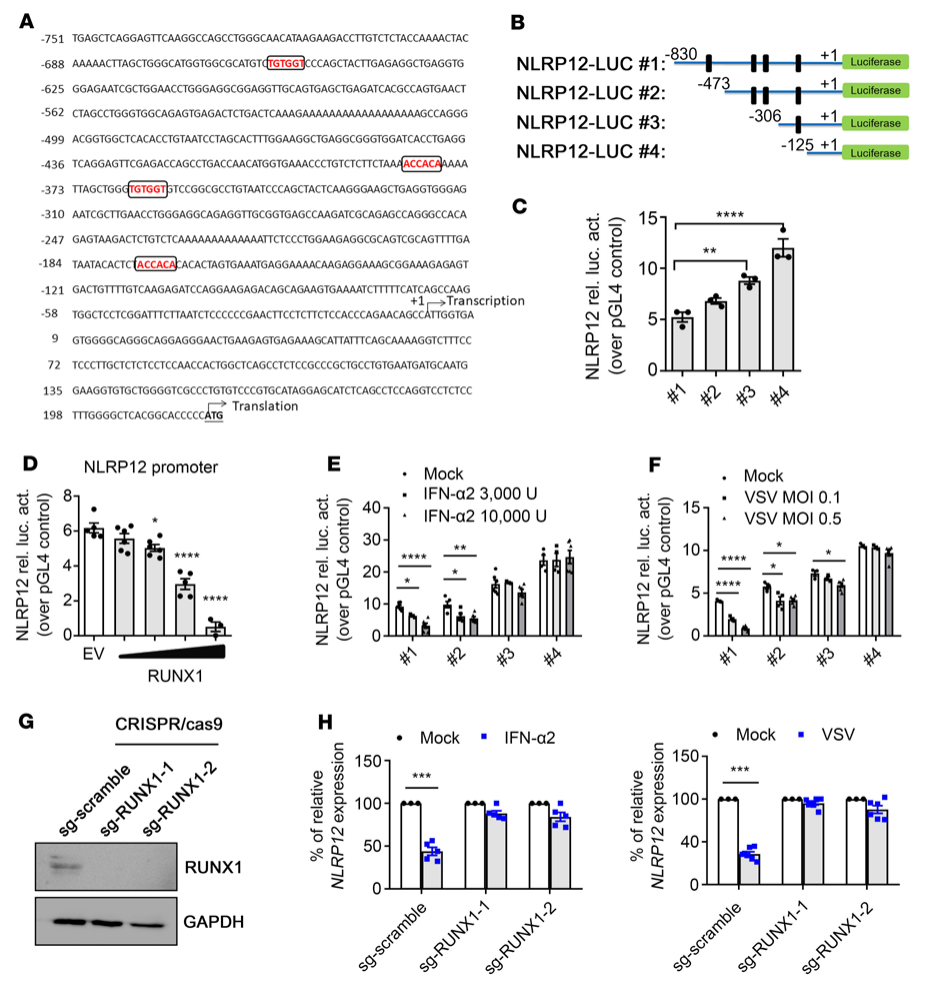
NLRP12 promoter contains RUNX1-binding sites. (**A**) Sequence of human NLRP12 promoter region from –751 to +222 bp. Letters in boxes denote binding sequences for RUNX1. (**B**) Schematic representation of NLRP12 promoter luciferase reporter constructs. For promoter analysis, an 830 bp length of NLRP12 promoter was cloned into pGL4-vector to drive luciferase reporter expression (NLRP12-Luc#1). Deletion constructs of NLRP12 promoter cloned into pGL4 vector are shown. Vertical lines are denoted as the RUNX1-binding motif on the NLRP12 promoter. (**C**) HEK293T cells were transfected with NLRP12-Luc#1 to NLRP12-Luc#4 plasmid and the internal control plasmid. Relative luciferase activity (rel. luc act.) was determined at 24 hours after transfection. (**D**) HEK293T cells transfected with NLRP12-Luc#1 and empty vector (EV) (pCDNA3) or RUNX-encoding plasmid (pCDNA3/DDK-RUNX1; 30, 100, 300, 500 ng/sample) and cell lysates were subjected to measurement of luciferase activity at 24 hours. (**E** and **F**) Human HT1080 and HEK293T cells were transfected with NLRP12-Luc#1 to NLRP12-Luc#4 for 6 hours, followed by IFN-α2 or VSV stimulation. Luciferase assays were performed at 24 hours. (**G**) Knockout of RUNX1 in THP-1 cells by CRISPR Cas9/sgRNA. (**H**) THP-1 cells with scrambled sgRNA or sgRNA targeting RUNX1 were treated with IFN-α2 or infected with VSV for 8 hours. *NLRP12* expression was measured. (**C**–**F**) One-way ANOVA test (multiple samples to a control); (**H**) 2-tailed Student’s *t* test. Data are represented as mean ± SEM (*n* = 5). *P < 0.05; **P < 0.01; ***P < 0.001.

To investigate the molecular mechanism of *NLRP12* transcriptional regulation, potential transcriptional binding sites were screened within 800 bp on the NLRP12 promoter region using the JASPAR database for eukaryotic transcription factor (TF) binding profiles (https://jaspar.genereg.net/). A conserved sequence motif (5′-TGTGGT/ACCACA-3′), recognized by a TF known as runt-related transcription factor 1 (RUNX1) (22) was found to have occurred 4 times (Figure 2A). Therefore, we evaluated the role of RUNX1 in the regulation of *NLRP12* expression by generating luciferase reporter plasmid driven by 830 bp (pGL4: –830 to +90 *NLRP12*) of the NLRP12 promoter region containing 4 RUNX1-binding sites (NLRP12-Luc#1). The shorter NLRP12 promoter containing 3 to no RUNX1 binding sequences was also generated, and the constructs were denoted as NLRP12-Luc#2 to NLRP12-Luc#4 (Figure 2B). The plasmids were transfected into HEK293T cells to monitor NLRP12 promoter activity. The results showed that the more RUNX1-binding motifs in the NLRP12-Luc reporter plasmid, the lower the NLRP12 luciferase activity (Figure 2C). Additional evidence revealed that NLRP12 luciferase activity was suppressed by the RUNX1 plasmid in a dose-dependent manner (Figure 2D). Therefore, the binding of RUNX1 to the NLRP12 promoter has an inhibitory effect on *NLRP12* expression. We further determined whether the binding of RUNX1 to the NLRP12 promoter was involved in IFN-α2– and virus-mediated NLRP12 promoter suppression. Human HT1080 cells and HEK293T cells were transfected with NLRP12-Luc#1 to NLRP12-Luc#4, followed by IFN-α2 treatment or VSV infection. The luciferase activity in the cells containing NLRP12-Luc#1 was significantly reduced in response to IFN-α2 and VSV, while cells with NLRP12-Luc#4 exhibited activity that was similar to the activity observed in the mock cells (Figure 2, E and F). We confirmed the importance of RUNX1 in regulating *NLRP12* expression by deleting RUNX1 in THP-1 cells with the CRISPR-Cas9 system (Figure 2G). *NLRP12* expression was not affected by IFN-α2 or VSV in THP-1 cells without RUNX1 expression (sg-*RUNX1*), while reduced *NLRP12* expression was observed in THP-1 cells containing sg-*RUNX1* scramble (Figure 2, H and I). Together, these results unveiled a role of RUNX1 located at the NLRP12 promoter that is required to suppress the transcriptional activation of *NLRP12* under IFN-I stimulation or during virus infection.

### Increased binding of RUNX1 protein to the NLRP12 promoter in SLE PBMCs.

**Figure 3:**
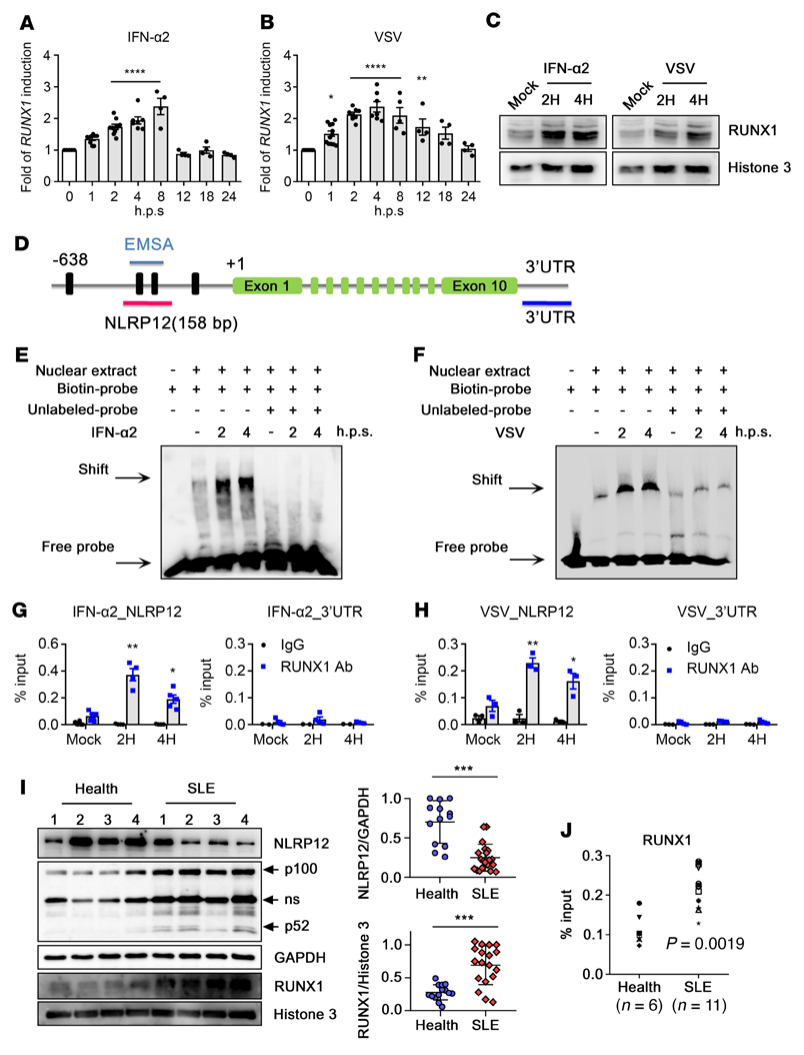
Increased binding of RUNX1 to the NLRP12 promoter region. (**A** and **B**) THP-1 cells treated with IFN-α2 or infected with VSV. *RUNX1* expression was determined at indicated time points. (**C**) Human CD14^+^ monocytes treated with IFN-α2 or infected with VSV. Levels of nuclear RUNX1 protein were analyzed with immunoblot. Histone 3 was used as a loading control. (**D**) Schematic representation of *NLRP12* gene from (+1). Vertical lines represent putative RUNX1-binding motifs. The locations of the EMSA probe and PCR products that are covered with the 2 RUNX1-binding sites or 3′ UTR of the NLRP12 promoter are shown. (**E** and **F**) Nuclear extracts obtained from CD14^+^ monocytes were treated with IFN-α2 or VSV. EMSA was conducted by using a biotin-labeled probe, and excess unlabeled probe was used to compete for this binding to validate the binding specificity. (**G** and **H**) CD14^+^ monocytes treated with IFN-α2 or VSV for 2 and 4 hours followed by a ChIP assay. (**I**) Lysates from CD14^+^ monocytes of healthy donors (*n* = 14) and SLE patients (*n* = 18) were subjected to immunoblot analysis. Representative image and quantitative densitometry are shown. (**J**) PBMCs from healthy donors and SLE patients (*n* = 11) were collected for ChIP analysis. (**A**–**H**) One-way ANOVA (multiple samples to the mock control); (**I**) 2-tailed Student’s *t* test; (**J**) Mann-Whitney *U* test. Data are represented as mean ± SEM. *P < 0.05; **P < 0.01; ***P < 0.001.

*RUNX1* expression was upregulated by IFN-α2 treatment and virus infection. Its expression peaked at 8 hours and returned to the basal level at 12 hours after IFN-α2 treatment in THP-1 cells (Figure 3A). VSV infection caused a longer induction of *RUNX1* expression than IFN-α2 treatment (Figure 3B). The level of nuclear RUNX1 protein was quickly increased after IFN-α2 stimulation and VSV infection in human CD14^+^ monocytes, which was in accordance with its transcription level (Figure 3C). Therefore, we explored whether the increase in RUNX1 protein levels enhanced its binding to the NLRP12 promoter. EMSA and ChIP assays were conducted to validate the binding of RUNX1 to the NLRP12 promoter after stimulation. For the EMSAs, nuclear extracts from human CD14^+^ monocytes were incubated with a DNA probe containing 2 RUNX1-binding sequences according to the NLRP12 promoter sequence (Figure 3D). There was a basal level of DNA-protein complexes in the nuclear extracts in the untreated monocytes (Figure 3, E and F; Lane 2), and increased levels of DNA-protein complexes were observed at 2 to 4 hours after IFN-α2 stimulation and VSV infection. This interaction was interfered with by unlabeled oligonucleotides, indicating binding specificity between the nuclear extract and specific DNA fragments. The binding of RUNX1 protein to the NLRP12 promoter was confirmed with a ChIP assay. RUNX1-containing DNA-protein complexes were pulled down with RUNX1 Abs, and the DNA product was assessed by quantitative PCR (qPCR) analysis with the primers amplifying RUNX1-binding sites on the NLRP12 promoter (Figure 3D). The results revealed specific binding of RUNX1 protein to the NLRP12 promoter. The levels of RUNX1-bound DNA were higher than those in the mock group, suggesting the significant enhancement of RUNX1 protein recruitment after stimulation (Figure 3, G and H). Importantly, SLE patient–derived monocytes expressed lower levels of NLRP12, but higher levels of RUNX1 protein than in healthy monocytes (Figure 3I, compiled data), and the elevated RUNX1 protein level was able to enhance the binding of RUNX1 protein to the *NLRP12* promoter. It is worth noting that SLE monocytes exhibited constitutive noncanonical NF-κB activation with the spontaneous processing of p100 into p52, implying that the elevated levels of immune signaling in patient-derived monocytes may correlate with the lower NLRP12 protein levels. ChIP data revealed significantly higher levels of RUNX1 protein binding to the *NLRP12* promoter in SLE patient–derived PBMCs than in healthy PBMCs (Figure 3J), which explains why RUNX1-mediated transcriptional suppression causes lower *NLRP12* expression in SLE patients.

### HDAC is involved in the IFN-I–mediated transcriptional suppression of NLRP12 expression.

**Figure 4:**
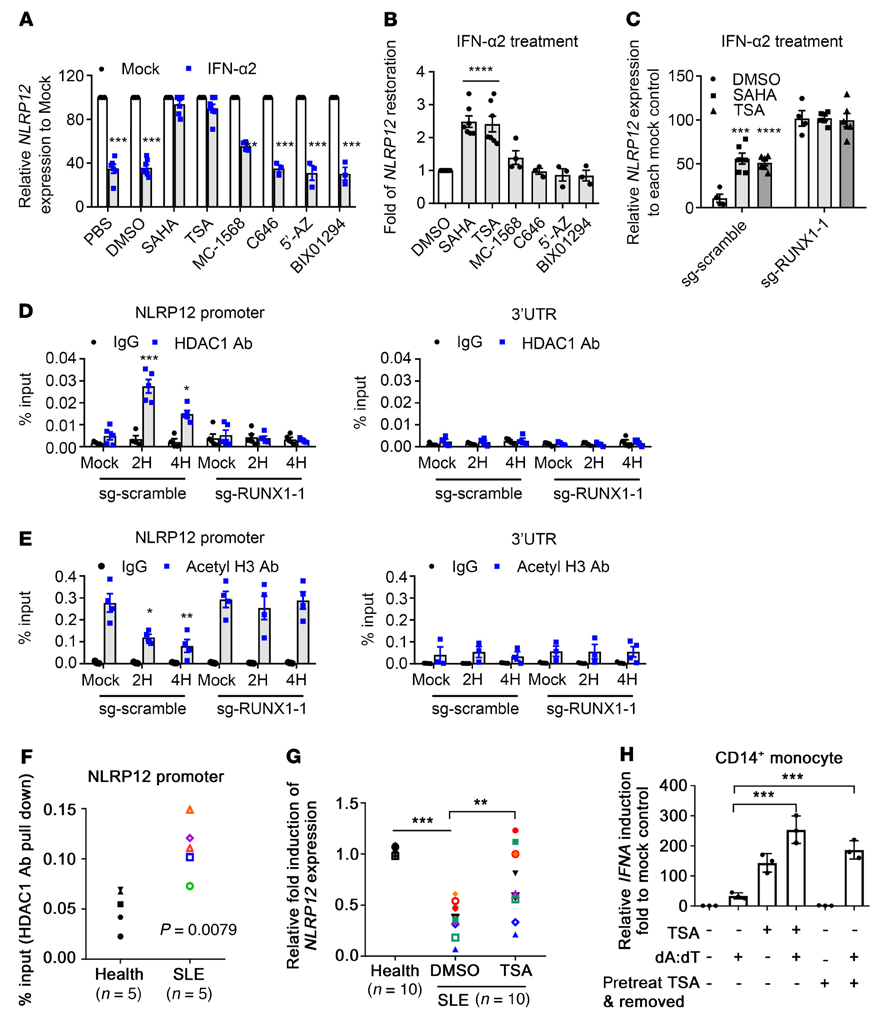
HDAC is involved in IFN-I–mediated transcriptional suppression of *NLRP12* expression. (**A** and **B**) THP-1 cells were preincubated with inhibitors for 30 minutes followed by treating cells with IFN-α2 for 6 hours. *NLRP12* expression was measured. (**B**) Restoration of *NLRP12* expression was calculated by setting *NLRP12* expression in IFN-α2–treated cells (DMSO) as 1. Relative *NLRP12* expression in the presence of inhibitors to DMSO group was measured. (**C**) THP-1/sg-scramble and THP-1/sg-RUNX1 were preincubated with TSA and SAHA and then treated with IFN-α2. *NLRP12* expression was measured. (**D** and **E**) THP-1/sg-scramble and THP-1/sg-RUNX1 treated with IFN-α2 were collected for ChIP analysis, in which the DNA-protein complex was pulled down by using control IgG and Abs to HDAC1 and acetyl-histone 3. Region of NLRP12 promoter and 3′ UTR was amplified as in previous description. (**F**) PBMCs (*n* = 5) from healthy donors and SLE patients were collected for ChIP analysis using a control IgG and an Ab to HDAC1. Region of NLRP12 promoter was amplified. (**G**) PBMCs from SLE patients (*n* = 10) were treated with DMSO or TSA for 4 hours.*NLRP12* expression relative to healthy PBMCs (*n* = 10) was measured. (**H**) CD14^+^ monocytes (*n* = 3) were treated with TSA for the entire period or for 4 hours, and TSA was removed, cells were transfected with poly(dA:dT), and *IFNA* expression was measured at 16 hours. (**A**) Two-tailed Student’s *t* test; (**B**–**E**, **G**, and **H**) 1-way ANOVA (multiple samples to the DMSO, or mock, or healthy control); (**F**) Mann-Whitney *U* test. Data are represented as mean ± SEM (*n* ≥ 5). *P < 0.05; **P < 0.01; ***P < 0.001.

Epigenetic regulation has been linked to the onset of SLE progression and its maintenance (23). To assess whether epigenetic regulation is involved in the transcriptional suppression of *NLRP12* expression, we pretreated THP-1 cells with various inhibitors and then stimulated the cells with IFN-α2. The suppression of *NLRP12* expression induced by IFN-α2 was significantly reversed by SAHA and TSA, an inhibitor of histone deacetylase 1 (HDAC1), while MC1568 (HDAC2 inhibitor) induced a moderate reversal (Figure 4, A and B). Histone acetyltransferase, DNA methylation, and histone methyltransferase inhibitors do not affect IFN-α2–induced changes in *NLRP12* expression. These observations suggest that HDACs, particularly HDAC1, are involved in the IFN-α2–mediated transcriptional suppression of *NLRP12* expression. RUNX1 protein was involved in HDAC1-mediated *NLRP12* transcriptional suppression. Because HDAC1 inhibitors prevented the downregulation of *NLRP12* expression in IFN-α2–treated THP-1/sg-scramble cells, but not in THP-1/sg-*RUNX1*-1 cells (Figure 4C), we suggested that the RUNX1 protein was involved in HDAC1-mediated NLRP12 transcriptional suppression. Results from ChIP analysis revealed that HDAC1 recruitment was significantly increased after IFN-α2 treatment (Figure 4D). The increased association between HDAC1 and *NLRP12* promoter was accompanied by a decreased association between acetyl-histone 3 and the *NLRP12* promoter (Figure 4E). Importantly, IFN-α2 increased the binding of HDAC1 and reduced H3ac levels in the NLRP12 promoter in THP-1/sg-scramble cells, while these effects were abolished in THP-1/sg-RUNX1-1 cells (Figure 4, D and E), suggesting the necessity of RUNX1 in *NLRP12* transcriptional suppression via epigenetic regulation. Higher levels of the HDAC1-NLRP12 promoter association in SLE patient–derived PBMCs was observed (Figure 4F), implying that the recruitment of an epigenetic factor to the NLRP12 promoter caused *NLRP12* transcriptional suppression in SLE patient–derived PBMCs. The lower *NLRP12* expression in SLE patient–derived PBMCs was rescued by an HDAC1 inhibitor in vitro, which further supported this conclusion (Figure 4G). We assessed whether an HDAC1 inhibitor could restore the *NLRP12* expression that restricts the *IFNA* expression induced by nucleic acid. To our surprise, *IFNA* expression in CD14^+^ monocytes either pretreated with HDAC1 inhibitor or incubated with inhibitor for the entire period was not decreased, but was significantly increased (Figure 4H). Although HDAC1 inhibitors restored *NLRP12* expression, their impact on *IFNA* expression goes far beyond the regulation of *IFNA* expression by NLRP12. Therefore, the HDAC1 inhibitor is still risky as a potential treatment for correcting epigenome alterations in SLE patients due to its concomitant effect of augmenting the IFN signature in autoimmune diseases (23).

### NLRP12 inhibits innate immune sensor-mediated pathways to negatively regulate cytokine production in response to nucleic acid stimulation.

**Figure 5:**
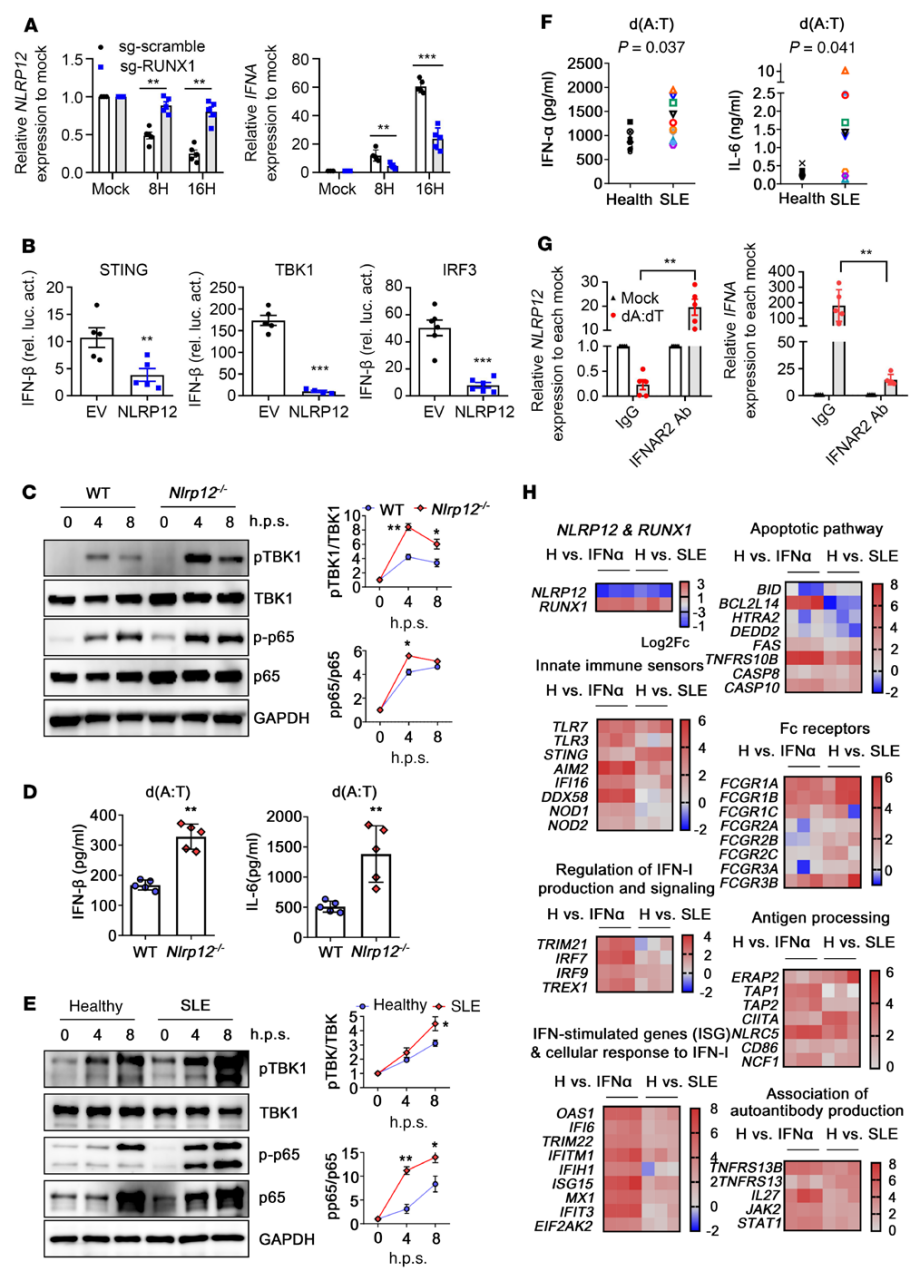
NLRP12 involved in innate immune signaling to negatively regulate cytokine production in response to nucleic acid stimulation. (**A**) THP-1/sg-scramble and THP-1/sg-RUNX1 were transfected with poly(dA:dT), and gene expression was measured. (**B**) HEK293T cells were cotransfected with 100 ng of IFN-β luciferase reporter, indicated plasmids (STING, TBK1 or IRF-3), and empty vector (pCDNA3) or NLRP12-encoding plasmid (pCDNA3/HA-NLRP12, 300 ng/sample). Luciferase assays were performed at 24 hours. (**C**) Mouse BMDCs from WT and *Nlrp12*^–/–^ mice were transfected with poly(dA:dT). Immunoblot was conducted and representative images and densitometry (relative to 0 hours) are shown. Bands were normalized with individual GAPDH. Ratio of phosphorylated protein to the total target protein was determined from 3 independent experiments. (**D**) WT and *Nlrp12*^–/–^ BMDCs were transfected with poly(dA:dT). Cytokine production was measured at 24 hours. (**E**) Human CD14^+^ monocytes from healthy donors and SLE patients were transfected with poly(dA:dT). Representative blots and densitometry are shown (*n* = 6). (**F**) CD14^+^ monocytes from healthy donors (*n* = 8) and SLE patients (*n* = 10) were transfected with poly(dA:dT). Cytokine production was measured at 24 hours. (**G**) Human CD14^+^ monocytes were preincubated with Abs to IFNAR2 for 30 minutes followed by transfecting cells with poly(dA:dT). Gene expression was analyzed at 16 hours. (**H**) Heatmap showing 2 DEG comparisons: (i) healthy monocytes versus IFN-α2–treated healthy monocytes (IFN-α), and (ii) IFN-α2–treated healthy monocytes versus SLE monocytes in each category. Color bars indicate scores of log_2_-fold change for each comparison. Data are represented as mean ± SEM. *P < 0.05; **P < 0.01; ***P < 0.001. Student’s *t* test (**A**–**E**, **G**); Mann-Whitney *U* test (**F**).

THP-1/sg-scramble cells transfected with poly(dA:dT) exhibited the RUNX1-dependent *NLRP12* transcriptional suppression that enhanced *IFNA* expression, while THP-1/sg-RUNX1 cells expressed lower *IFNA* expression in response to poly(dA:dT) stimulation (Figure 5A). We explored whether NLRP12 affects the immune signaling cascades driven by other sensors that regulate the production of IFN-I and proinflammatory cytokines. The luciferase reporter assay was conducted by cotransfecting the IFN-β promoter or NF-κB reporter plasmid with the NLRP12 plasmids plus the innate immune sensor or critical adaptor plasmid in HEK293T cells. The impact of NLRP12 expression in affecting luciferase activity was analyzed. We showed that overexpression of NLRP12 in HEK293T cells significantly attenuated the activity of the IFN-β promoter driven by STING, TBK1, and IRF-3 (Figure 5B), which involved the cGAS/STING pathway in driving the induction of IFN-I (24). NLRP12 also suppressed the activation of the IFN-β promoter and NF-κB promoter activated by IRAK1 and TRIF (Supplemental Figure 2, A and B), which are critical mediators downstream of the endosomal TLR7/9 and TLR3 signaling pathways (25). We validated the change in the phosphorylation of effectors that act downstream of those immune signaling cascades. The production of phosphorylated TBK1 (pTBK1) and NF-κB p65 (p-p65) was induced by 4 to 8 hours after poly(dA:dT) stimulation in WT bone marrow–derived DCs (BMDCs), while these signals were significantly higher in *Nlrp12^–/–^* BMDCs (Figure 5C). The differences in the levels of those phosphorylated effectors between WT and *Nlrp12^–/–^* BMDCs reflect the levels of IFN-α and IL-6 production (Figure 5D), and *Nlrp12^–/–^* BMDCs expressed higher levels of IFN-α and IL-6 induced by other types of nucleic acids (Supplemental Figure 2, C and D). We assumed that SLE patient–derived monocytes with much lower *NLRP12* expression levels might produce higher levels of cytokines than those obtained from healthy donors after nucleic acid stimulation. As expected, the basal levels (0 hours) of TBK1 and p65 were higher in patient-derived monocytes than in healthy monocytes, and poly(dA:dT) further induced higher levels of pTBK1 and p-p65 in SLE patient–derived monocytes than in healthy monocytes (Figure 5E). Ten SLE patients and 8 healthy donors were enrolled to assess cytokine production after ligand stimulation (Supplemental Table 3). With nucleic acid stimulation, patient-derived monocytes produced significantly higher IFN-α and IL-6 levels in response to poly(dA:dT), poly(I:C), and CL097 stimulation (Figure 5F and Supplemental Figure 2, E and F) These results implied that patient-derived monocytes are more susceptible to producing IFN-I and inflammatory cytokines under exposure to nucleic acids originating from cell debris, ICs, and pathogen infections, which further promote the development of the IFN-I signature and inflammation in SLE patients. In fact, these 10 patients all exhibited low to moderate disease activity with SLEDAI-2K of 6 or less. Despite their stable disease activity as sampled before this analysis, the hyperresponsive phenotype of their monocytes toward the production of IFN-I and IL-6 in response to extrinsic stimuli possibly indicates the fluctuation of disease activity due to the sustained low *NLRP12* expression. We investigated whether IFN receptor (IFNR) signaling was essential for the nucleic acid–induced transcriptional suppression of *NLRP12* expression. The levels of *NLRP12* expression in poly(dA:dT)-transfected human monocytes were not reduced in the presence of IFNAR2 blockade, while IFNR signaling exhibited a feed-forward effect on *IFNA* expression (Figure 5G).

### IFN-I–driven biological processes contribute to the pathogenesis of SLE.

IFN signature genes are highly expressed in the PBMCs of SLE patients and are associated with active disease progression (26). To determine whether the activation of pathways induced by IFN-α stimulation was similarly induced in SLE patient–derived monocytes, we conducted RNA-Seq analysis. Transcriptome gene set enrichment analysis (GSEA) was based on the hallmark gene set in healthy monocytes, IFN-α–treated healthy monocytes, and SLE patient–derived CD14^+^ monocytes. GSEA was used to compare the differentially expressed genes (DEGs) between (a) healthy monocytes versus (b) IFN-α–treated–healthy monocytes; and (a) healthy monocytes versus (c) SLE patient–derived monocytes. We found that the genes belonging to the IFN-α and IFN-γ response pathways as well as immune signal transduction pathways, such as TNF signaling via NF-κB and IL-6/STAT3, were enriched in IFN-α–treated and SLE patient–derived monocytes (Supplemental Figure 3, A and B), suggesting a resemblance of the previously observed impact of IFN-I in the monocytes of SLE patients. We also analyzed the differentially expressed genes (DEGs) using Ingenuity Pathway Analysis (QIAGEN) to assess their participation in different biological processes, in which genes relevant to the pathogenesis of SLE were grouped to generate the heatmap (Figure 5H). The gene expression of innate immune sensors was generally upregulated after IFN-α treatment, and *STING* expression was much higher in the SLE group than in the IFN-α–treated group. The expression of the ISGs and the DEGs belonging to IFN-I signaling and cellular response to IFN-I were mostly upregulated in IFN-α–treated monocytes and SLE patient–derived monocytes. The genes that regulate the apoptotic pathway have been reported to be associated with SLE, in which *FAS*, *TNFRS10B*, *CASP8*, and *CASP10* expression levels were all increased in IFN-α–treated and SLE monocytes, while the levels of the other measured genes were not identical between the IFN-α and SLE groups. This finding suggests that specific apoptotic pathways participating in the pathogenesis of SLE were regulated by IFN-I. Finally, the levels of DEGs in monocytes involved in antigen processing, Ab production, and survival signals for plasma cells, including *TNFSF13B*, *TNFSF13* and *IL-27*, were mostly increased in both IFN-α–treated monocytes and SLE patient–derived monocytes. These results indicate that IFN-I–derived biological processes are considerably involved in the pathogenesis of SLE, particularly in autoantibody production. Consequently, IFN-α induced changes in *NLRP12* and *RUNX1* expression in monocytes linked with the patterns observed in SLE patient–derived monocytes, suggesting the role of NLRP12 in the IFN-I–derived pathogenesis of SLE.

### Increased levels of inflammation and IFN-I production lead to a greater immune response in pristane-treated NLRP12-deficient mice.

**Figure 6:**
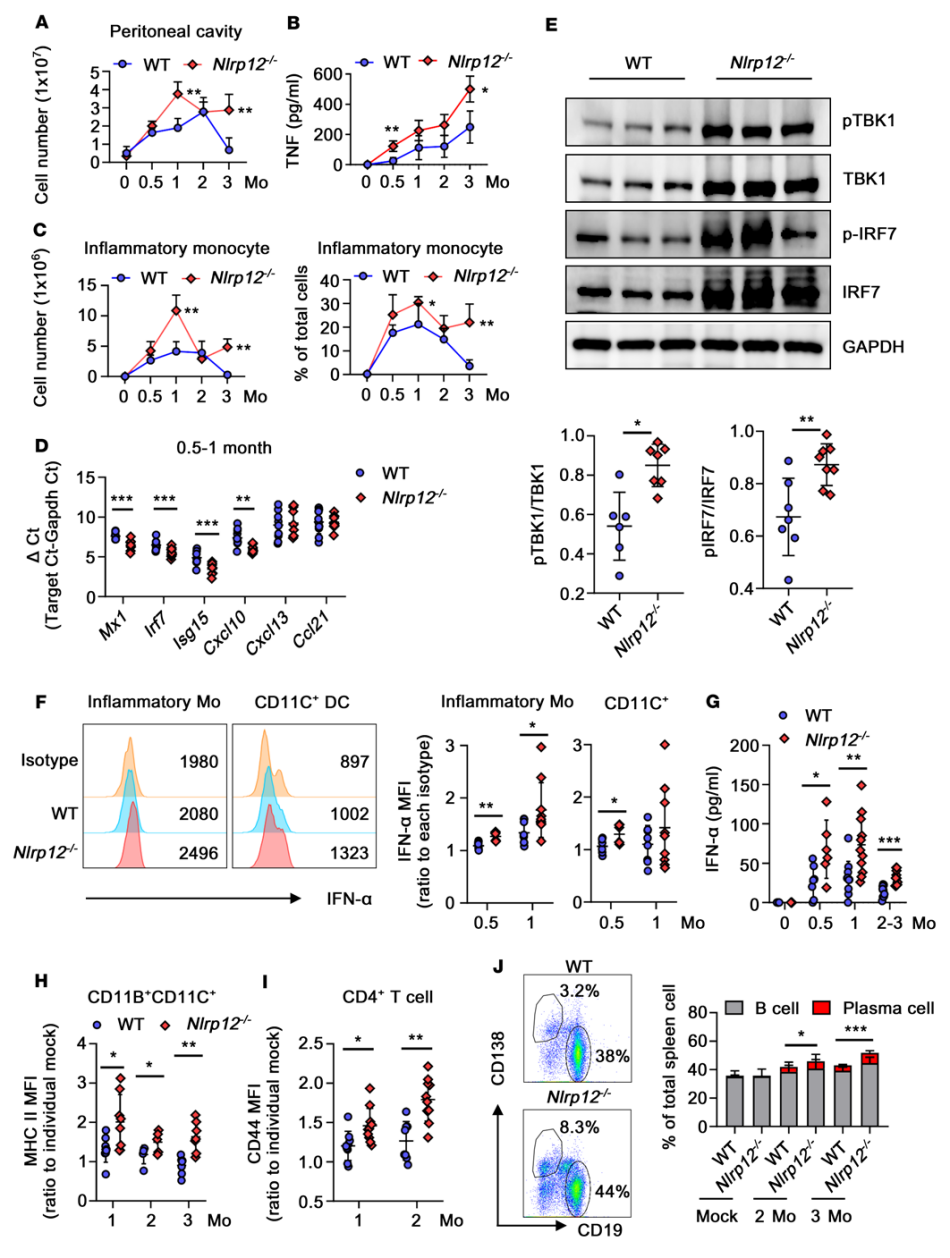
NLRP12-deficient mice display greater immune response and higher IFN-I production in response to pristane injection. Mice receiving pristane injection were sacrificed at indicated time points. (**A**) Numbers of infiltrated cells in the peritoneal cavity were recorded. (**B**) Amount of TNF in peritoneal lavage fluid was measured by cytometric bead array (CBA) analysis. (**C**) The numbers and percentages of recruited inflammatory monocytes were measured by FACS. (**D**) The gene expression of IFN signatures in PECs was measured; data were displayed with ΔCt, which stands for absolute gene expression level. (**E**) Representative immunoblots of the PECs at 1 month after injection and quantitative densitometry are shown. (**F**) IFN-α expression in peritoneal Ly6C^hi^CCR2^hi^CD11B^+^CD11C^–^F4/80^+^ inflammatory monocytes and CD11B^+^CD11C^+^F4/80^+^Ly6C^–^Ly6G^–^ DCs was measured by FACS. Histogram from a representative sample at 0.5 months after injection (left). Data compilations (*n* = 6~9, right) were expressed as the MFI relative to the corresponding isotype control. (**G**) IFN-α amounts in peritoneal lavage fluid were measured by ELISA. (**H**) Relative MHC class II (MHC II) expression of splenic CD11C^+^ DCs and (**I**) relative CD44 expression of splenic CD4^+^ T cells were measured by FACS. (**J**) Representative dot blots of the proportion of CD19^+^- and CD138^+^-expressing cells in splenocytes from mice at 3 months after injection (left). Data (*n* = 8 each, right) show percentages of B cell (CD3^–^CD19^+^) and plasma cell (CD3^–^CD19^–^CD138^+^) populations. Percentages of plasma cells were compared between WT and *Nlrp12*^–/–^ mice and analyzed with 2-tailed Student’s *t* test. (**F**–**I**). Two-tailed Student’s *t* test. Data are represented as mean ± SEM. *P < 0.05; **P < 0.01; ***P < 0.001.

Mice that receive peritoneal administration of pristane, a hydrocarbon oil (2,6,10,14-tetramethylpentadecane), exhibit TLR7 signaling–dependent peritoneal inflammation, autoantibody production, IFN-I production, and GN, which are similar to the processes occurring in SLE patients (27). Pristane administration causes peritonitis in mice with an early influx of immune cells into the peritoneal cavity. *Nlrp12^–/–^* mice exhibited aggravated inflammation with high levels of cell infiltration and higher levels of TNF, CCL2, and IL-6 in the peritoneal lavage fluid throughout the entire period, while delayed cell recruitment and dramatic reductions in cell levels were noted in WT mice (Figure 6, A and B, and Supplemental Figure 4A). Among the cytokine-producing cells, the levels of inflammatory monocytes (Ly6C^hi^CCR2^hi^CD11B^+^CD11C^–^F4/80^+^), which constitute approximately 20%–30% of total peritoneal cells (PECs), gradually increased to a peak during the first month (Figure 6C). *Nlrp12^–/–^* mice had significantly higher counts of peritoneal inflammatory monocytes than WT mice, and these were maintained until the late stage due to higher levels of CCL2 production in the peritoneum. Similarly, *Nlrp12^–/–^* mice showed higher levels and sustained recruitment of peritoneal CD11C^+^ DCs (CD11B^+^CD11C^+^F4/80^+^Ly6C^–^Ly6G^–^) and granulocytes (CD11B^+^CD11C^–^F4/80^+^Ly6C^int^Ly6G^hi^) in the peritoneum (Supplemental Figure 4, B and C). During the late stage, the numbers of residential macrophages (F4/80^hi^CD11B^+^CD11C^–^Ly6C^–^Ly6G^–^) were gradually restored in WT mice, whereas inflammatory monocytes persisted in *Nlrp12^–/–^* mice accompanied by long-lasting peritonitis (Supplemental Figure 4D). All of these observations indicated that the augmented and long-lasting inflammatory conditions were preferentially maintained under NLRP12-deficient conditions. Persistent recruitment of inflammatory monocytes is attributable to the induction of CCL2 production in an IFN-I signaling–dependent manner (28). Therefore, we assessed the expression levels of the general genes involved in the IFN signature in WT and *Nlrp12^–/–^* PECs. Because we were unable to harvest enough PECs for RNA preparation from the mock-treated mice, we used ΔCt to indicate the absolute gene expression level of each target. The lower the ΔCt value, the higher the gene expression. Among the genes we analyzed, the expression levels of *Mx1*, *Isg15*, *Irf7*, and *Cxcl10* in *Nlrp12^–/–^* PECs were significantly higher than those in WT PECs (Figure 6D). Moreover, the PECs from pristane-treated WT and *Nlrp12^–/–^* mice expressed different levels of effectors, including TBK1, pTBK1, IRF7, and pIRF7, which act downstream of the immune signaling cascade, suggesting higher levels of IFN-α expression in *Nlrp12^–/–^* PECs (Figure 6E). Inflammatory monocytes and CD11C^+^ DCs were the major IFN-α–producing cells in PECs, with a much lower number of pDCs (CD3^–^B220^+^CD11C^int^PDCA1^+^, data not shown). IFN-α production was induced as early as 2 weeks after pristane stimulation; it was sustained for 1 month (Figure 6F) and gradually decreased from 2 to 3 months (Figure 6G). Then the PECs displayed IFN signatures instead (Supplemental Figure 4E). Peritoneal inflammatory monocytes and DCs obtained from *Nlrp12^–/–^* mice exhibited higher IFN-α levels than those obtained from WT mice (Figure 6F), which is in concordance with the higher IFN-α levels measured in the peritoneal fluids (Figure 6G). The frequency of inflammatory monocytes, granulocytes, and CD11C^+^ DCs in the spleen, in addition to the peritoneal cavity, of *Nlrp12^–/–^* mice was markedly higher than that in WT littermates (Supplemental Figure 5A). Surface MHC class II expression was induced in WT splenic CD11C^+^ DCs during the first month of pristane administration, which gradually decreased at from 2 to 3 months. In contrast, *Nlrp12^–/–^* DCs expressed relatively higher surface MHC class II after pristane administration, and high MHC class II expression was maintained longer (Figure 6H). CIITA, a master TF of MHC class II genes, was transcriptionally upregulated in either IFN-I–treated CD14^+^ monocytes or SLE monocytes (Figure 5H), which supported the idea that the IFN-signaling cascade led to DC activation in SLE patients and *Nlrp12^–/–^* mice. Moreover, CD44 expression was enhanced in splenic CD4^+^ T cells in pristane-treated mice, and its levels continued to increase and remained high in *Nlrp12^–/–^* mice (Figure 6I). The higher CD44 expression in CD4^+^ T cells was associated with a greater proportion of pDCs in *Nlrp12^–/–^* mice (Supplemental Figure 5B) because their capacity in IFN-I production contributes to DC activation and the optimal response of CD4^+^ T cells (29). Splenic CD19^+^ B cells exhibited higher MHC class II and CD40 levels in pristane-treated *Nlrp12^–/–^* mice than in WT mice (Supplemental Figure 5C), which was accompanied by a higher proportion of T follicular cells (CD3^+^CD4^+^CXCR5^+^PD1^+^) in *Nlrp12^–/–^* mice than WT mice, and thus this was most likely mediated by greater T cell/B cell interplay (Supplemental Figure 5D). The levels of B and plasma cells in mouse spleens were increased after pristane administration. There was a continual increase in the levels of those cells in *Nlrp12^–/–^* mice, with 44% B cells and 8% plasma cells in total splenocytes, but not in WT mice during the third month (Figure 6J). Collectively, these results indicate that the greater levels of inflammation and IFN-I production led to a more potent immune response in pristane-treated *Nlrp12^–/–^* mice.

### NLRP12 deficiency enhances autoantibody formation and immune cell infiltration in a pristane-induced lupus-like mouse model.

**Figure 7:**
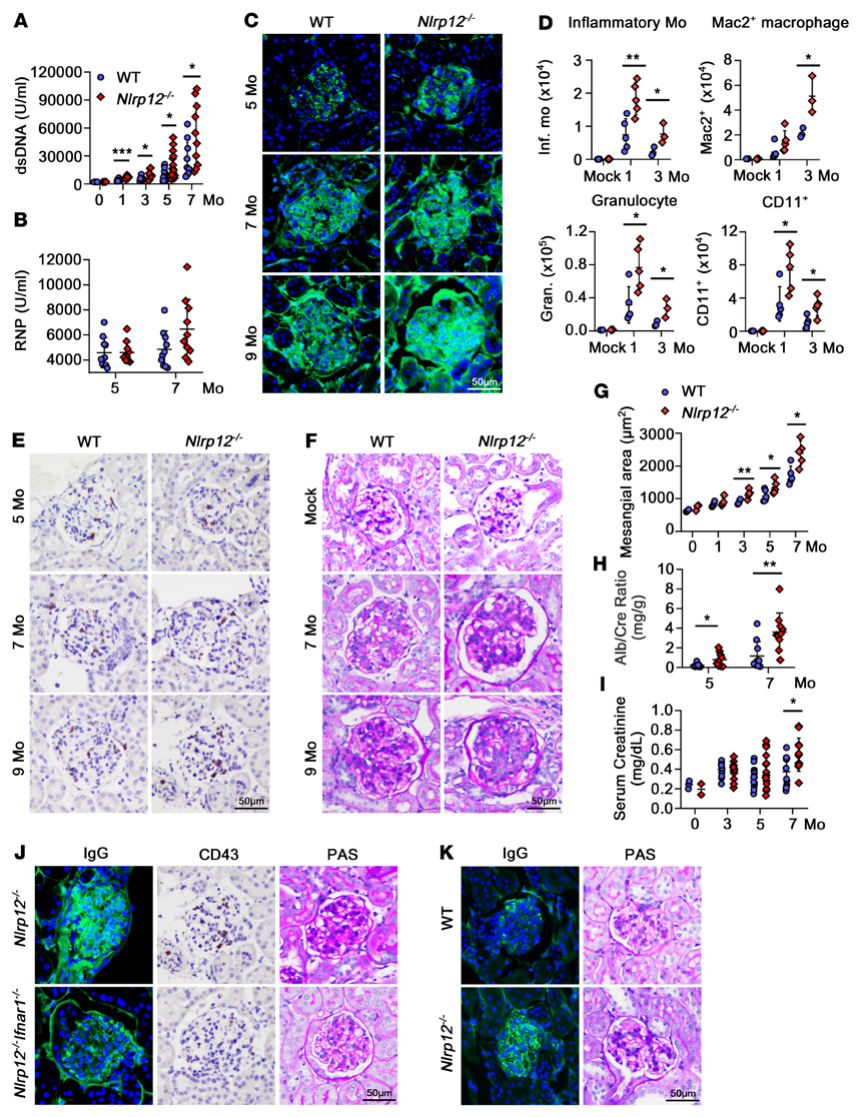
NLRP12-deficient mice presented more severe disease course in the pristane-induced lupus-like model. (**A**) Levels of serum anti-dsDNA Abs and (**B**) anti-RNP Abs in WT and *Nlrp12^–/–^* mice were measured. (**C**) Immunofluorescence staining of IgG from kidney sections at fifth, seventh, and ninth months in WT and *Nlrp12^–/–^* mice. (**D**) FACS analysis of population of myeloid immune cells in kidney including inflammatory monocytes, Mac2^+^ macrophages, granulocytes, and CD11C^+^ cells. (**E**) Representative CD430- and (**F**) PAS-stained WT and *Nlrp12^–/–^* glomeruli. (**G**) Calculation of the average of mesangial area in glomerulus using the MetaMorph Imaging System (Molecular Devices). (**H**) Measurement of mouse urine ACR. (**I**) Measurement of mouse serum creatinine. (**J**) Representative IgG-, CD43-, and PAS-stained *Nlrp12^–/–^Ifnar1^–/–^* and *Nlrp12^–/–^* glomeruli at the ninth month. (**K**) Representative IgG- and PAS-stained glomeruli from WT and *Nlrp12^–/–^* mice treated with IMQ for 5 weeks. Two-tailed Student’s *t* test. Data are represented as mean ± SEM. *P < 0.05; **P < 0.01; ***P < 0.001. Scale bars: 50 μm.

The pristane-treated *Nlrp12^–/–^* mice had a higher frequency of B–to–plasma cell transition accompanied by an increasing amount of anti-dsDNA Abs in serum compared with WT mice from the first to the seventh month (Figure 7A). An increasing number of anti-RNP Abs were observed during the seventh month (*P* = 0.08, Figure 7B). The extent of IgG deposition and C3 fixation in the glomerulus and in the tubules was very different in WT and *Nlrp12^–/–^* mice (Figure 7C and Supplemental Figure 6, A–C). During the fifth month after challenge, WT mice showed spotty IgG deposition in the glomerulus, while *Nlrp12^–/–^* mice exhibited expanded mesangial deposition (Figure 7C). In the following months, *Nlrp12^–/–^* mice exhibited more IgG deposition, which enabled the progression to global GN with a stronger intensity of IC deposition in the mice. Intensive infiltration of CD11B^+^ myeloid cells mainly around the tubules, not the glomerulus, was observed during the first month, which gradually reduced in the third month in WT mice (Supplemental Figure 6D). Higher levels of CD11B^+^ cell infiltration associating with greater IL-6 expression were noted in *Nlrp12^–/–^* mice during the third month (Supplemental Figure 6E), and the increase of fibrin deposition persisted in *Nlrp12^–/–^* mice (Supplemental Figure 6F). These kidney CD11B^+^ cells mainly comprised inflammatory monocytes, granulocytes, Mac2^+^ macrophages, and CD11C^+^ DCs and were recruited into the tubule area during the first month; their levels reduced after acute inflammation (Figure 7D and Supplemental Figure 7, A and B). In *Nlrp12^–/–^* mice, the frequency of these inflammatory myeloid cells was significantly higher than that in WT mice (Figure 7D), and CD11B^+^ cells also expressed higher levels of IFN-α (Supplemental Figure 7). Therefore, the existence of competent myeloid cells with IL-6– and IFN-α– producing capacity contributes to prolonged inflammation that causes tubule damage in pristane-treated *Nlrp12^–/–^* mice.

### NLRP12-deficient mice exhibited significant glomerular monocyte infiltration and IgG deposition that exacerbated the progression of GN.

LN-presenting GN is mainly mediated by the glomerular deposition of ICs and complement components and is a hallmark of SLE pathology that constitutes a major morbidity in patients. The pristane-induced lupus-like mouse model triggered significant GN, in which TLR/IFNAR and IFN-I/IFNAR signaling, immune cell infiltration, and IC-induced inflammation were all involved (27). IFN-I production induced by pristane injection increases the expression levels of ISGs, such as *Ccl2* and *Cxcl-10*, to recruit myeloid cells, particularly inflammatory monocytes (28). In fact, the influx of monocytes plays a substantial role in the pathogenesis of LN (30, 31), particularly their influx into the kidney, which is considered an indicator of GN (32). We observed that *Nlrp12^–/–^* mice had greater levels of Ly6C^hi^ monocyte infiltration in the glomerular basement membrane and mesangium (Supplemental Figure 8A). Interestingly, the patrolling monocytes, including either CD11b^+^CX3CR1^hi^Ly6C^lo^ (31) or CD11b^+^CD43^hi^ (30) in mice, which are orthologs of human nonclassic CD14^–^CD16^++^ monocytes (33), were shown to be involved in LN in mouse and human, respectively (30, 31, 34). We observed an increase in the levels of CD43^+^ cells in the glomeruli from the third month, and it was predominant in *Nlrp12^–/–^* mice after the fifth month (Supplemental Figure 8B). The infiltration of CD43^+^ cells peaked during the seventh month, and this high level was maintained until the ninth month (Figure 7E) in *Nlrp12^–/–^* mice. It has been reported that the deposited glomerular ICs are able to recruit circulating nonclassic monocytes via Fcγ receptor IIIA from the blood flow in a mouse model (31). Human nonclassic monocytes in the stage IV LN with capillary IC deposition are accompanied by the expression of endothelial CX3CL1, which is a chemoattractant for patrolling monocytes (34). CD43^+^ patrolling monocytes are responsible for recognizing the nucleic acids and driving TNF production (30, 31). Therefore, we speculated that *Nlrp12^–/–^* mice exhibit higher levels of glomerular IC deposition that promotes inflammatory cells, particularly CD43^+^ cell–mediated pathogenesis in LN (Figure 7E). During the fifth month, histological analysis revealed expansion of periodic acid–Schiff (PAS) staining in WT mice, but more mesangial proliferation, enlarged tufts, surrounding tubule epithelium effacement, and tubular dilation (Supplemental Figure 8C) in *Nlrp12^–/–^* mice were observed. During the seventh month, marked expansion of the mesangial area with concomitant obliteration of capillaries was observed in *Nlrp12^–/–^* mice, which exhibited sclerotic changes during the ninth month (Figure 7F). We quantified the mesangial area in each glomerulus (Supplemental Figure 9), and *Nlrp12^–/–^* mice exhibited significantly higher mesangial area than WT mice after the third month (Figure 7G). An increased urine albumin/creatinine ratio (ACR) was observed from the fifth to the seventh month, and *Nlrp12^–/–^* mice had a higher ACR than WT mice (Figure 7H). These findings were associated with damage to glomeruli that caused higher serum creatinine levels in *Nlrp12^–/–^* mice during the seventh month (Figure 7I), indicating more severe deterioration of kidney function in pristane-treated *Nlrp12^–/–^* mice. Because NLRP12 deficiency amplified the IFN signature and disease severity in mice, we validated whether IFNR signaling was critical for NLRP12-mediated regulation using *Nlrp12^–/–^Ifnar1^–/–^* mice. Lower levels of IgG deposition and CD43^+^ cell indwelling were observed in *Nlrp12^–/–^Ifnar1^–/–^* mice than in pristane-treated *Nlrp12^–/–^* mice during the ninth month. The glomerulus displayed much less mesangial expansion and few wire-loop capillaries in *Nlrp12^–/–^Ifnar1^–/–^* mice (Figure 7J). These observations confirmed that aberrant IFNR signaling is one of the essential factors in the context of the NLRP12-derived pathogenesis of lupus, and blockade of IFNAR might be a potential strategy for the treatment of SLE patients with intrinsically low *NLRP12* expression. Another lupus model driven by TLR7 ligand involving topical imiquimod (IMQ) stimulation was used (35). *Nlrp12^–/–^* mice had higher levels of IgG deposition and mesangial expansion in the glomerulus after 5 weeks of IMQ stimulation than WT mice (Figure 7K). This strengthened the involvement of NLRP12 in the regulation of the IFNR signaling–mediated pathogenesis. In summary, the deposition of IgG/ICs in the glomerulus causes a series of inflammatory reactions, including immune cell recruitment, in which myeloid cells with aberrant expression of proinflammatory cytokines and IFNs disturb resident renal cells and eventually lead to renal fibrosis (36).

### NLRP12 deficiency enhances the expansion of lymphocytes in mice carrying the lpr mutation, which exacerbates the progression of GN.

**Figure 8:**
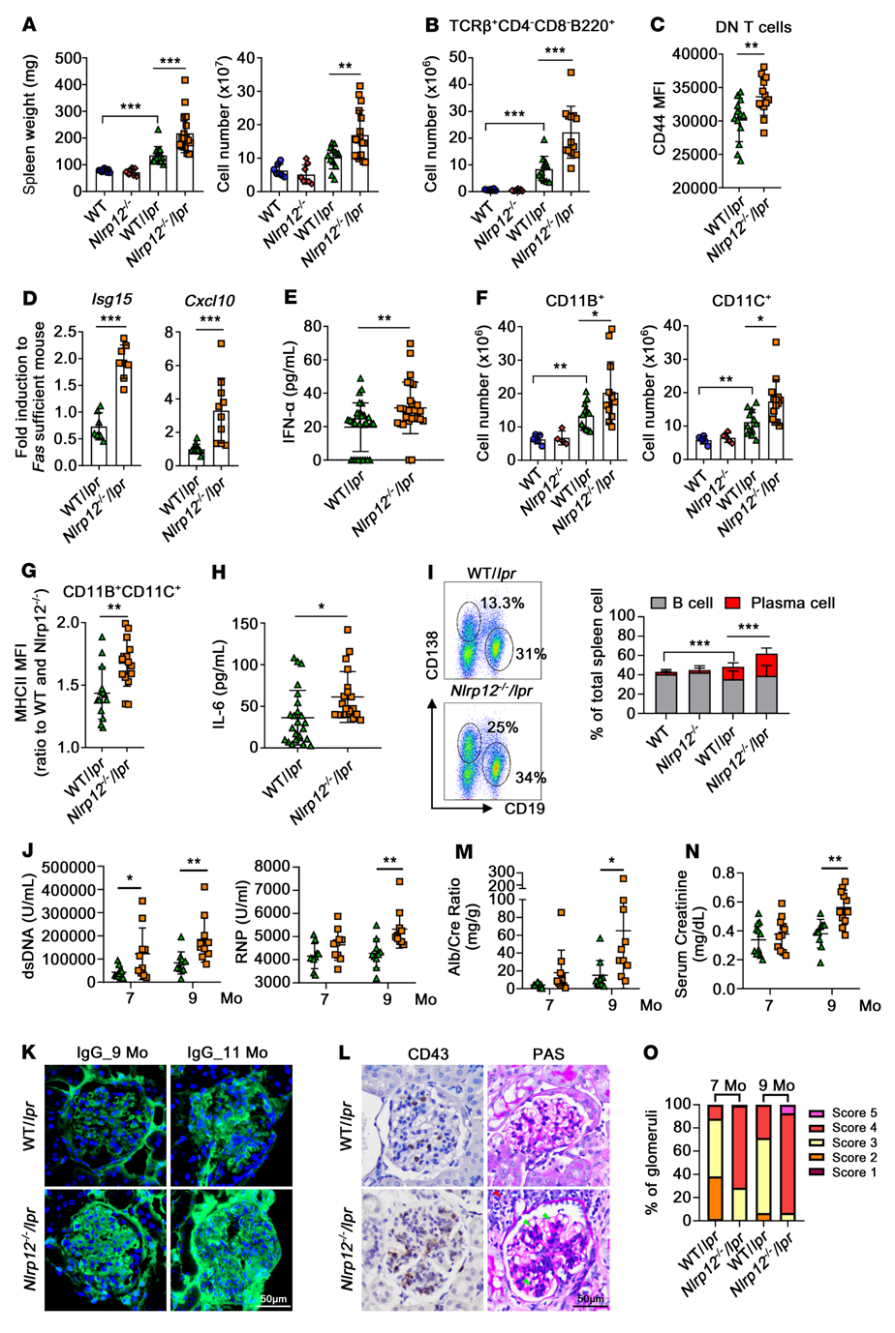
NLRP12 deficiency enhances expansion of immune cells in *Fas^lpr^* mice that exacerbates the progression of GN. (**A**) Weight of spleens and total number of splenocytes were recorded. (**B**) Number of splenic TCRβ^+^CD3^+^CD4^–^CD8^–^ B220^+^ (DN) T cells and (**C**) expression levels of CD44 in DN T subset were analyzed by FACS. (**D**) Expression of *Isg15* and *Cxcl10* in splenocytes. (**E**) Amounts of serum IFN-α were measured by ELISA. (**F**) Numbers of splenic CD11B^+^and CD11C^+^ cells and (**G**) expression levels of MHC class II on CD11B^+^CD11C^+^ cells were analyzed by FACS. (**H**) Amounts of serum IL-6 were measured by ELISA. (**I**) Representative dot blots of the proportion of CD19^+^- and CD138^+^-expressing cells in splenocytes from mice. Compiled data (WT and *Nlrp12^–/–^*, *n* = 10; WT/*lpr* and *Nlrp12^–/–^*/*lpr* mice, *n* = 20) showed percentages of B cell (CD3^–^CD19^+^ CD138^–^) and plasma cell (CD3^–^CD19^–^CD138^+^) populations. (**J**) Levels of serum anti-dsDNA and anti-RNP Abs from WT/*lpr* and *Nlrp12^–/–^*/*lpr* mice were measured. (**K**) Representative IgG-stained WT/*lpr* mice and *Nlrp12^–/–^*/*lpr* glomeruli. (**L**) Representative CD43- and PAS-stained WT/*lpr* mice and *Nlrp12^–/–^*/*lpr* glomeruli at ninth month. Scale bars: 50 μm. (**M**) Measurement of urine ACR and (**N**) serum creatinine. (**O**) Percentages of glomerulus index distribution in WT/*lpr* and *Nlrp12^–/–^*/*lpr* mice. (**A**–**E**, **F**, and **G**) Shown are data from 28- to 30-week-old mice, *n* = 10–18; (**E** and **H**) 28- to 40-week-old mice, *n* = 20–28. (**A**, **B**, and **F**) One-way ANOVA; (**C**–**E** and **G**–**N**) 2-tailed Student’s *t* test. Data are represented as mean ± SEM. *P < 0.05; **P < 0.01; ***P < 0.001.

Mice homozygous for the lymphoproliferation spontaneous mutation (*Fas^lpr^*) showing systemic autoimmunity are used for studying SLE. To validate the role of NLRP12 in a lupus model driven by genetic mutation, we generated autoimmune-prone mice by crossing B6.MRL-*Fas^lpr/lpr^*/J mice with *Nlrp12^–/–^* mice. The offspring of those mice were *Nlrp12^–/–^* and *Fas^lpr^* homozygous (referred to as *Nlrp12^–/–^*/*lpr*) and *Nlrp12^+/+^Fas^lpr/lpr^* (referred to as WT/*lpr*). Although *Fas^lpr^* mice with a C57BL/6J background exhibited severe symptoms of lupus by 40 to 42 weeks (37), WT/*lpr* mice exhibited splenomegaly with a significantly increased number of splenocytes at 26 to 28 weeks (Figure 8A). *Nlrp12^–/–^*/*lpr* mice displayed an even higher number of splenocytes and greater degree of splenomegaly than WT/*lpr* mice. The Fas mutation dysregulates the lymphocyte population to disturb peripheral tolerance, leading to lymphadenopathy and splenomegaly primarily involving the TCRβ^+^CD3^+^CD4^–^CD8^–^ B220^+^ (double-negative [DN]) T cell subset in humans and mice with Fas deficiency (38). We found that not only CD4^+^ and CD8^+^ T cells, but also splenic DN T cells, were significantly increased in mice carrying *lpr*, and *Nlrp12^–/–^*/*lpr* mice had a greater number of DN T cells than WT/*lpr* mice (Figure 8B). The DN T cells in *Nlrp12^–/–^*/*lpr* mice expressed higher levels of surface CD44 than those in WT/*lpr* mice (Figure 8C). Therefore, increased surface CD44 expression in DN T cells in *Nlrp12^–/–^*/*lpr* mice may correlate with greater immune activation and the potential mobilization of effector T cells at sites of inflammation in a lupus model driven by the *lpr* mutation. Interestingly, the accumulation of the DN T subset caused by Fas deficiency occurs in an eomesodermin-dependent (Eomes-dependent) manner. T cell–specific deletion of Eomes prevents accumulation of DN T in Fas-deficient mice, and overexpression of Eomes enables expansion of unusual CD8-related subsets (38). It is worth noting that IFN-I can upregulate Eomes expression to promote the development of unconventional CD8^+^ T cells (39). The expression levels of *Isg15* and *Cxcl10* (Figure 8D) and the levels of serum IFN-α (Figure 8E) were higher in *Nlrp12^–/–^*/*lpr* mice than in WT/*lpr* mice. All these findings provide an explanation for why *Nlrp12^–/–^*/*lpr* mice exhibited higher levels of accumulation of DN T subsets in the spleen. The number and activation status of myeloid cells in WT/*lpr* mice were higher than those in WT and *Nlrp12^–/–^* mice (Figure 8F), and *Nlrp12^–/–^*/*lpr* mice showed significant expansion and activation of myeloid cells (Figure 8G), which are associated with an enhanced capacity of antigen presentation and inflammatory milieu, such as the augmented serum levels of IL-6 (Figure 8H). WT/*lpr* mice had a significantly greater proportion of plasma cells than WT mice (1.9% versus 12.9%); *Nlrp12^–/–^*/*lpr* mice had a higher level of plasma cells than WT/*lpr* mice (Avg., 12.9% vs. 23.2%, Figure 8I). Serum anti-dsDNA and anti-RNP Abs were detected during the seventh and ninth months with a tendency toward increasing titers, *Nlrp12^–/–^*/*lpr* mice presented higher anti-dsDNA Ab titers than WT/*lpr* mice, and *Nlrp12^–/–^*/*lpr* mice also had higher anti-RNP Ab titers during the ninth month (Figure 8J). In WT/*lpr* mice, IgG deposition increased from the ninth to the eleventh month, but there was a fulminant expansion of IgG deposition over the glomerulus in *Nlrp12^–/–^*/*lpr* mice during the ninth month, which developed into heavy deposition during the eleventh month (Figure 8K). The pattern of infiltration of CD43^+^ cells aligned with the trend of glomerular IgG deposition, and *Nlrp12^–/–^*/*lpr* mice had higher levels of patrolling monocytes in the glomerulus than WT/*lpr* mice, which was associated with mesangial expansion, wire-loop formation of capillaries, and extensive immune cell infiltration during the ninth month (Figure 8L). *Nlrp12^–/–^*/*lpr* mice exhibited progressive sclerosis during the eleventh month (Supplemental Figure 10A). Unlike in WT/*lpr* mice, the higher urine ACR (Figure 8M) and serum creatinine levels (Figure 8N) observed in *Nlrp12^–/–^*/*lpr* mice indicated serious GN during the ninth month. Due to the heterogeneity of the severity presentation in each glomerulus, we assessed over 200 glomeruli from *Nlrp12^–/–^*/*lpr* and WT/*lpr* and scored the severity (Supplemental Figure 10B). Glomeruli in *Nlrp12^–/–^*/*lpr* mice mainly scored 4, while those in WT/*lpr* mice mainly exhibited a score of 3 during the seventh and ninth months (Figure 8O). The *Nlrp12^–/–^*/*lpr* mice exhibited further progression to glomerular sclerosis, with 7.3% of glomeruli showing complete sclerosis during the ninth month (score 5), revealing the acceleration of disease activity under NLRP12 deficiency. In summary, the deficiency of NLRP12 in *lpr* mice exacerbates disease progression in the typical lupus-prone model, causing a substantial shifting of plasma cells in the spleen and promoting the generation of more autoantibodies and leading to subsequent glomerular damage.

## Discussion

This work reveals that a negative feedback loop between NLRP12 protein and IFN-I production plays a key role in the pathological progression of LN. The central role of IFN-I in SLE is remarkable. The consistently low *NLRP12* expression in SLE patient–derived PBMCs results in excessive immune activation, which critically drives the initiation and progression of the disease. The factors that drive the downregulation of *NLRP12* expression can be varied. We show that virus infection, nucleic acid, and IFN-α treatment markedly reduced *NLRP12* expression by RUNX1-dependent epigenetic regulation, as observed in SLE patient–derived PBMCs. Interestingly, IFN-α–containing serum can also reduce *NLRP12* expression in THP1 cells, but this reduction is restricted to sera containing IFN-α levels higher than 20 pg/mL and has a dose-dependent effect. It is worth noting that patients with detectable circulating IFN-α levels had significantly higher SLEDAI-2K than patients without detectable serum IFN-α levels (*P* = 0.004), suggesting that SLE patients with higher disease activity remain at risk for prolonged IFN-I signature gene expression and inflammation due to lower *NLRP12* expression. Additionally, the expression of IFN-I signature genes was not exactly compatible with the elevated IFN-α levels observed in SLE patient–derived serum and was observed only in some patients (12). Serum data from our cohort are in accordance with a previous study in which we detected only 12 IFN-α–positive sera among 85 serum samples. Although there were only 12 patients with detectable circulating IFN-α levels, most of the patients exhibited lower *NLRP12* and higher *IFNA* levels in their PBMCs. The reduction in NLRP12 levels could be a consequence of previous IFN-α exposure; thus, the IFN signature was present and persisted, even though circulating IFN-α was not always detected in patients at the time of sampling. Lupus patients with lower *NLRP12* levels not only had increased levels of IFN-I, but also presented with deteriorated clinical parameters (Figure 1). Given that the IFN signature was observed in patients with higher disease activity (26), we showed that SLEDAI-2K and *NLRP12* expression in PBMCs exhibited a trend of inverse correlation (Supplemental Figure 11A). Patients in group 1 (relative *NLRP12* expression <0.43) had a higher SLEDAI-2K index than those in group 2 (Supplemental Figure 11B). Interestingly, patients with LN presented with lower *NLRP12* levels in their PBMCs than patients without LN (Supplemental Figure 11C). Therefore, low *NLRP12* expression might be a specific marker associated with LN. Notably, monocytes from lupus patients diagnosed with low-to-moderate disease activity under stable conditions produced higher amounts of IFN-α and IL-6 than healthy monocytes after nucleic acid ligand stimulation. Therefore, the paradoxical condition “steady-like but unstable disease” contributes to the vulnerability of lupus patients with disease activity that is obscured beneath the well-controlled index when minor infections, such as upper respiratory infections, occur. Currently, clinical scores, such as those based on SLEDAI or the British Isles Lupus Assessment Group Index (BILAG) (40), are widely used in clinical evaluation. These indices provide a systemic evaluation of lupus patients, but the lack of serological evaluation is the current shortfall of this approach, and thus these methods may not reflect disease conditions in time. The lack of an ideal marker that corresponds with disease burden dynamically and precisely is a current challenge (41). To address the gap between serological markers and disease activity, we speculated that NLRP12 would be used as a serologic biomarker for “treat-to-target” in lupus patients because its expression level is increased in patients under adequate treatment. The imminent flare in lupus patients would be eliminated with adequate treatments to elevate *NLRP12* expression, especially when patients stayed at low disease activity or remission of clinical index. Traditional treatment of SLE is based on the combination of corticosteroids, hydroxychloroquine, azathiopurine, and mycophenolate mofetil. Growing evidence has emphasized the beneficial role of properly controlling the IFNR-signaling cascade to mitigate the inflammatory condition observed in lupus. In 2021, the FDA approved the use of anifrolumab, an IFN-I receptor blocker, as a novel biologic in treating lupus (42). The success of this drug in treating lupus by interfering with the IFN signature confirmed that both innate and adaptive immunity concurrently contribute to the pathogenesis of lupus. In addition, anifrolumab ameliorates overall lupus activity with a larger effect on both low and high IFN-I signature groups and was able to maintain the low IFN signature status in these 2 groups based on the MUSE trial (43). Another study reported that the baseline expression levels of IFN-related genes, such as *EPSTI1*, *IFI44 L*, *LY6E*, *OAS3*, and *RSAD2*, are sufficient to predict flares and outcomes in SLE patients (44), but they may not be feasible for use in daily clinical practice. Low *NLRP12* expression was apparently observed in SLE patients, and this low expression was associated with the increased expression levels of IFN signature genes. Thus, *NLRP12* expression could act as a “turnkey marker” of the IFN signature both theoretically and practically.

Genetic susceptibility and environmental exposure are two major factors involved in the initiation and development of lupus disease. In this study, we evaluated the role of NLRP12 in the pathogenesis of lupus in 2 mouse models: pristane-induced lupus-like and *Fas* mutation–mediated lupus-prone models. The pristane model involves an environmental trigger, such as infection, that induces acute inflammation, immune cell infiltration, and the production of proinflammatory cytokines and IFN-I. In contrast, B6.MRL-Fas^lpr^/J mice exhibit less production of proinflammatory cytokines and IFN-I than mice in the pristane model, but their genetic background drives lymphoid hypertrophy that disrupts immune tolerance and causes chronic inflammation. Pristane administration triggered systemic inflammation and aggravated IFN-I expression in mice, and IFN-I further drove the expression of CCL2 and CX3CL1 to recruit Ly6C^hi^ inflammatory monocytes and CD43^+^ patrolling monocytes into the kidney, respectively. NLRP12 deficiency augments the production of proinflammatory cytokines and IFN-I, which promote the recruitment of pathogenic monocytes, accelerate disease progression, and prolong kidney inflammation. In addition, the potent immune response in *Nlrp12^–/–^* mice leads to the generation of circulating autoantibodies and subsequent glomerular IC deposition that collaboratively exacerbate tissue damage and glomerulus fibrosis. Unlike in the pristane model, B6.MRL-Fas*^lpr^* (WT/*lpr*) mice with profound lymphoproliferation lost immune tolerance in different ways, but eventually developed lupus over time. These mice exhibited higher levels of anti-dsDNA Abs than WT mice that received pristane stimulation due to a dramatic B–to–plasma cell transition. Glomerular IC deposition in WT/*lpr* mice was mainly surrounded by capillaries inside the glomerulus; thus, wire-loop deposition and sclerotic changes were observed, while pristane-treated WT mice showed spotty IgG deposition in the glomerulus with more mesangial deposition. The different patterns of IC deposition in these 2 models may be associated with diverse immune pathways and undefined mechanisms. *Nlrp12^–/–^*/*lpr* mice showed robust IC in the glomerulus accompanied by the profound recruitment of CD43^+^ patrolling monocytes unlike in WT/*lpr* mice (Supplemental Figure 12). Although IFN-I expression was not dominant in the *Fas* mutation model, NLRP12 deficiency in mice carrying lpr increased the levels of systemic IL-6 and IFN-α as well as the numbers of DN T cells in the spleen to accelerate disease progression. IFNR signaling is critical for NLRP12-mediated immune regulation because attenuated IgG deposition and impaired recruitment of CD43^+^ monocytes in pristane-treated *Nlrp12^–/–^Ifnar1^–/–^* mice were observed (Supplemental Figure 12). The consistent trend of elevated anti-dsDNA Ab titers, urine ACR, glomerular IC deposition, and the subsequent mesangial expansion and pathologic change as well as increased serum creatinine levels over time in NLRP12-deficient mice in the 2 lupus models confirmed its role in the pathogenesis of lupus disease.

## Methods

### Human subjects.

SLE patients and age-matched healthy controls provided peripheral blood for analysis with clinical manifestations. Antinuclear Abs were detected with Fluoro-Kit (DiaSorin Inc.). Anti-dsDNA Abs were quantified with ELISA via BINDAZYME (The Binding Site Ltd.). The specific IgGs against SSA/Ro, Sm, and RNP were quantified with a solid phase (ImmunoCAP 100, Phadia AB).

### Mouse and animal models.

WT C57BL/6J (The Jackson Laboratory) and *Nlrp12^–/–^* (18) mice at 12 week of age were injected intraperitoneally with a single dose of 500 μl pristane (2,6,10,14-tetramethylpentadecane). No significant differences in biochemical index and histopathological change were observed between sexes. The *Nlpr12^–/–^Ifnar1^–/–^* mice were generated by crossing *Nlrp12^–/–^* and *Ifnar1^–/–^* mice (C57BL/6J, gift of Lee Chien Kao, National Taiwan University, Taipei, Taiwan). For the IMQ model, in 10-week-old mice, 5 mg of IMQ cream (Aldara, 5% IMQ) was applied topically to the right ear 3 times a week for 5 weeks to induce a lupus-like phenotype. The B6.MRL-Fas^lpr^/J mice were purchased from Jackson Laboratory. *Nlrp12^–/–^*/*lpr* were generated by crossing B6.MRL-Fas^lpr^/J with *Nlpr12^–/–^* mice (*Nlrp12^–/–^Fas^lpr/lpr^*, Supplemental Figure 13). Female mice were used in the lupus-prone model.

### Experimental procedures.

Sample preparation, mRNA analysis, ligand stimulation, luciferase reporter assay, EMSA, ChIP, immunoblot analysis, flow cytometry analysis, histopathologic assessment, and measurement of mouse autoantibodies and cytokines are described in detail in Supplemental Methods. RNA-Seq raw data files can be found in the NCBI’s Gene Expression Omnibus database (GEO GSE218492).

### Statistics.

Data were analyzed by SPSS, version 21.0, and Prism software (version 8.0, GraphPad Software). For mouse and cellular experiments, data are represented as the mean ± SEM and were evaluated by 2-tailed Student’s *t* test or 1-way ANOVA. For human samples, parametric data were evaluated by 2-tailed Student’s *t* test, and nonparametric data were evaluated by the Mann-Whitney *U* test. Spearman’s regression was performed for correlation of variables. *P* < 0.05 was considered statistically significant.

### Study approval.

Human studies were approved by the Institutional Review Boards of Taipei Veterans General Hospital (no. 2018-02-011AC, no.2022-01-017BC). Informed, written consent was obtained before sampling. All animal experiments were approved by the Institutional Animal Care and Use Committee of National Yang Ming Chiao Tung University (IACUC 1070612, 1091011).

## Author contributions

YPT provided clinical data, conducted patient recruitment, designed and performed experiments, performed data analysis, and contributed to writing the manuscript. FYT, CWC, SYC, ICC, PHW, and WTC performed experiments. MHC and CYT conducted patient recruitment. PCC provided the detailed protocol and reagents for ChIP analysis and offered the instruments for this study. YCY performed pathological studies. BOA performed bioinformatics analysis. CSW and CYT provided technical support. STC designed and performed experiments, performed data analysis, and contributed to writing the manuscript.

## Supplementary Material

Supplemental data

## Version 1 02/01/2023

Electronic publication

## Version 2 04/25/2023

Updated Figures 1, 5, and affiliations
