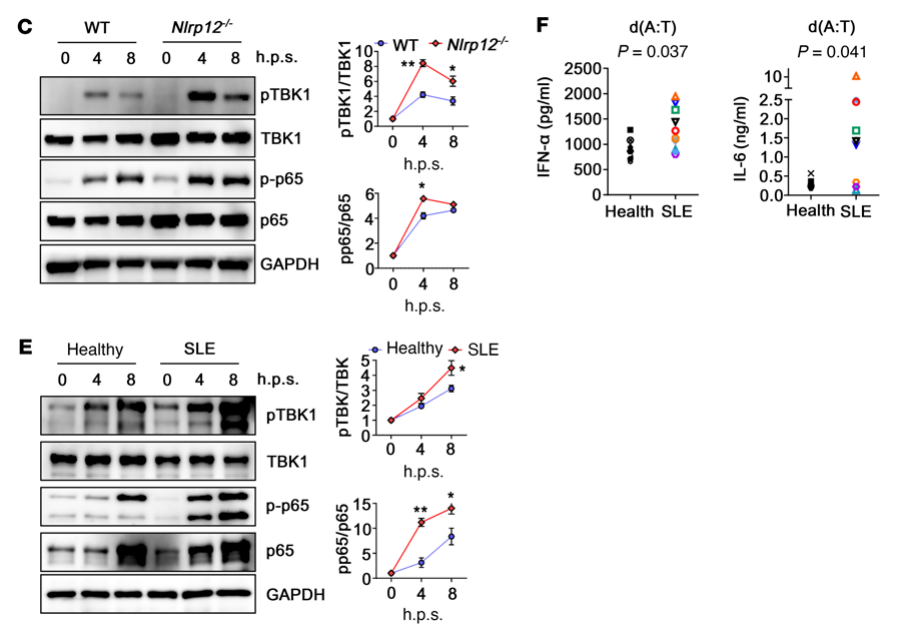# NLRP12 is an innate immune checkpoint for repressing IFN signatures and attenuating lupus nephritis progression

**DOI:** 10.1172/JCI171350

**Published:** 2023-05-01

**Authors:** Yen-Po Tsao, Fang-Yu Tseng, Chih-Wei Chao, Ming-Han Chen, Yi-Chen Yeh, Babamale Olarewaju Abdulkareem, Se-Yi Chen, Wen-Ting Chuang, Pei-Ching Chang, I-Chun Chen, Pin-Hsuan Wang, Chien-Sheng Wu, Chang-Youh Tsai, Szu-Ting Chen

Original citation: *J Clin Invest*. 2023;133(3):1–19. https://doi.org/10.1172/JCI157272

Citation for this corrigendum: *J Clin Invest*. 2023;133(9):171350. https://doi.org/10.1172/JCI171350

Figures 1, F, J, and Q, reported incorrect *P* values. For Figure 5, C and E, the *x* axis labels were incorrect and h.p.s. was not defined. Figure 5F was missing *P* values. In addition, Dr. Szu-Ting Chen’s affiliations were incorrect. The correct figure parts, definition, and affiliations list are below.

h.p.s., hours post stimulation. 

Yen-Po Tsao,^1,2,3^ Fang-Yu Tseng,^4^ Chih-Wei Chao,^1,4^ Ming-Han Chen,^2^ Yi-Chen Yeh,^5^ Babamale Olarewaju Abdulkareem,^6^ Se-Yi Chen,^7,8^ Wen-Ting Chuang,^1^ Pei-Ching Chang,^9^ I-Chun Chen,^1^ Pin-Hsuan Wang,^1^ Chien-Sheng Wu,^10^ Chang-Youh Tsai,^2^ and Szu-Ting Chen^1,4,6,11^

## Figures and Tables

**Figure:**
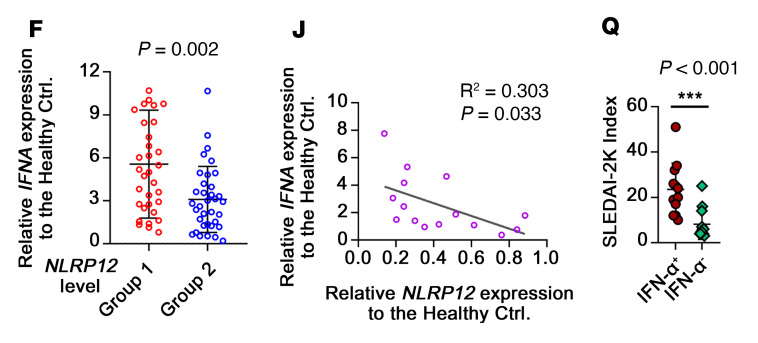


**Figure:**