# Supplementary material for: NLRP12 is an innate immune checkpoint for repressing IFN signatures and attenuating lupus nephritis progression
Source: J Clin Invest. 2023 Feb 1;133(3):e157272. doi: 10.1172/JCI157272 (PMC9888378; doi:10.1172/JCI157272)
Supplement: Supplemental data [file jci-133-157272-s009.pdf]

Supplemental Methods

RNA Isolation and mRNA Analysis

Total RNA was extracted from cell or tissue lysate using TRIzol (Thermo Scientific). cDNA was generated from total RNA with ReverAid first strand cDNA Synthesis Kit (Cat.K1622, Thermo Scientific, Inc.). Quantitative RT-PCR was performed on a StepOnePlus 7 Real-Time PCR System with SYBR Green PCR Master Mix (Cat. BIO-98005, Thermo Scientific, Inc.). Sequences of the oligonucleotides were summarized in Supplementary Table 4.

Expression plasmids

The Myc-DDK-tagged full-length RUNX1 expression plasmid obtained from OriGene Technologies, human RUNX1 cDNA (accession no. NM_001001890) was inserted into a pCMV6-Entry vector. A sequence containing 920 bp of NLRP12 promoter was cloned into pGL4 luciferase reporter vector (Promega) and called NLRP12-Luc#1 (pGL4:-830~+90). The primers were 5'- GGTACCTACGTGAGGTCC-3' and 5'-AGATCTGCAAGGGAGGAAAG-3'. To generate truncated NLRP12 promoter with the deletion of RUNX1 binding sites, the forward primer sequences containing KpnI sites for cloning were used, pGL4: -473~+90 (NLRP12-Luc#2); 5'-GGTACCTGTAATCCTAGCAC-3'; pGL4: -306~+90 (NLRP12-Luc#3); 5'-GGTACCGGGAGAATCGCTT-3'; pGL4: -125~+90 (NLRP12-Luc#4); 5'-GGTACCGGAAAGAGAGTGA-3'; a common reverse primer contains BglII site was used 5'-AGATCTGCAAGGGAGGAAAG-3'.

Generation of RUNX1 Knockout cell line with CRISPR/Cas9

Insert RNA sequences for CRISPR/Cas9 were designed at web site (http://crispr.mit.edu/) provided by the National RNAi Core Facility (Academia Sinica, Taiwan). Insert oligonucleotides for human RUNX1 gRNA #1 and #2 are 5'-GATGAGCGAGGCGTTGCCGC-3' and 5'-GTGAGCGAGGCGTTGCCGCT-3', respectively. The complementary oligonucleotides for guide RNAs (gRNAs) were annealed and cloned into pAll-Cas9.Ppuro vector (Academia Sinica, Taiwan). RUNX1-gRNA was co-transfected into HEK293 T with packaging plasmids pMDG and pCMVdelta R8.9 to make lentivirus. THP1-RUNX1-gRNA and THP1-RUNX1-gRNA stable-lines with homozygous property were selected and maintained in the 2 μg/ml of puromycin containing medium.

Cell Preparation

Human PBMCs were isolated from blood by standard density-gradient centrifugation

with Ficoll-Paque (Amersham Biosciences). CD14$^+$ cells were purified from PBMCs by high-gradient magnetic sorting, using VarioMACS technique with anti-CD14 microbeads (Miltenyi Biotec GmbH). Mouse BMDCs were isolated from femurs and tibias and cultured in RPMI1640 complete medium supplemented with 10% (v/v) FCS, L-Glutamine, pen/strep and 40 ng/mL recombinant mouse GM-CSF (R&D Systems) for 9 days (1). Human monocytic cell line: THP-1 (ATCC® TIB-202) and human fibrosarcoma HT-1080 (ATCC® CCL-121™) were obtained from the ATCC.

Ligand Stimulation and Virus Infection

THP-1, human CD14$^+$monocytes and mouse BMDC were applied for the ligand stimulation. Poly (dA:dT), poly (dC:dG), LMW poly(I:C) were suspended in LyoVec™ transfection reagent (InvivoGen) in a concentration of 0.01 mg/ml for 15 min and then was transfected into cells (1μg ligand for 1×10^5 cells). Human CD14$^+$ monocytes or THP-1 were infected with Vesicular stomatitis Virus-Indiana strain (ATCC® VR-1419™) or human herpesvirus 1 (ATCC® VR-1493™) at the multiplicity of infection (MOI) of 0.1. For cytokine treatment, human CD14$^+$monocytes or THP-1 cells were treated with IFNα2 (3,000 U) for 4 or 8 hours as mention in legends. In the study of the inhibitors to the epigenetic regulation, 3 μM or10 μM those inhibitors were used to treat the cells, followed by stimulating cells with ligands.

Dual-luciferase reporter assay

HEK293T (3×10^5 cells in a 24-well plates) were transfected using lipofectamine™ 2000 (Cat.11668019, Thermo Scientific, Inc.). Ten ng of pRT-TK Renilla luciferase reporter plasmid and 100 ng of firefly luciferase reporter plasmids were co-transfected with indicated plasmids. For NLRP12 promoter, 50 ng of pRT-TK Renilla luciferase reporter plasmid and 500 ng of NLRP12 promoter firefly luciferase reporter plasmids (pLG4) were co-transfected with RUNX1 plasmids. Luciferase activity was measured 24 h after transfection using the Dual-Glo Luciferase Assay System (Cat.E2920, Promega, Inc.). Renilla luciferase reporter plasmid as an internal control was transfected in all the setting of luciferase reporter assay.

Immunoblot analysis

Cells were lysed in buffer containing 20 mM HEPES and KOH (pH 7.5), 150 mM NaCl, 1 mM EDTA, 0.5% NP-40, 10% glycerol, 10 mM pyrophosphate, supplemented with complete proteases inhibitors (Roche). Lysates were resolved by SDS-PAGE and transferred onto PVDF membrane. Membranes were probed with Abs against NLRP12 (Cat.675202; BioLegend), RUNX1 (Cat.23980; abcam) and visualized using ECL (Thermo Fisher Scientific). The following reagents are all from Cell Signaling

Technology: Abs against Histone 3 (#3972), IRF7 (#4920), phospho-IRF7 (#5184), TBK1 (#3013), phospho-TBK1 (#5483), NFκB p65 (#3034), phospho-NFκB p65 (#3033), NFκB2p100/p52 (#4882).

EMSA

Cytoplasmic and nuclear fractions were extracted using NE-PER nuclear and cytoplasmic extraction reagents. Gel shift assay was conducted by LightShift® Chemiluminescent EMSA kit (ThermoFisher). Eight μg of nuclear extract was incubated with serial of reaction components. Biotin-labeled DNA probe (hot probe) containing consensus RUNX1 binding sequences (500 fmol) was added to the reaction mixed followed by adding a non-labeled DNA probe (cold probe, 30 pmol) to compete the DNA (hot probe)-protein interaction. The DNA-protein complexes were resolved on a non-denaturing polyacrylamide gel prior to chemiluminescent graphic imaging. DNA probe was generated by annealing the oligonucleotides and its complementary strands. The sequence of oligonucleotide: 5'CCTGTCTCTTCTAAAACCACAAAAATTAGCTG GGTGTGGTGT-3', and the consensus RUNX1 binding site is underlined.

Chromatin immunoprecipitation (ChIP)

Cells (3×10^7) per reaction were fixed with formaldehyde to crosslink the protein-DNA complexes, and DNA in lysed cells was sonicated to lengths of 500-1000 bp in a Bioruptor (UCD-200TM; Diagenode). Rabbit IgG (cat.17180; Abcam), antibodies to RUNX1 (Cat.23980; Abcam), acetylated Histone H3 (Cat.06–599; Millipore) and HDAC1 (Cat.06–720; Millipore) were used for immunoprecipitation. The DNA-protein complexes were sequentially washed and eluted, then the immunoprecipitated DNA was purified with the Thermo GeneJET PCR purification kit (Cat. K0702; Thermo). SYBR® Green quantitative qPCR was performed and values were expressed as a percent of total input DNA. Primers to amplify NLRP12 promoter we re: 5'-CCCTGTCTCTTCTAAAACCACAAAA-3' (forward) and 5'-GTCTTACTCTGTGGCCCTGGCTCTG-3' (reversed) and the amplified product contains two RUNX1 binding sites. Primers amplifying an unrelated site at 3'-UTR of *NLRP12* gene were used to confirm the specificity.

RNA-seq analysis.

$CD14^+$ monocytes were sorted from healthy donors and patients with MACS® isolation kit. Healthy $CD14^+$ monocytes were treated with or without IFNα2 (3000 U) for 4 hours, and RNA was extracted using Qiagen miRNeasy Mini Kit. RNA integrity and RNA integrity number (RIN) are assessed using an RNA ScreenTape® and the TapeStation

Analysis Software A.02.01 SR1 and validated for high-throughput RNA sequencing (oligo d(T) enriched approach). RNA-seq was performed by GENEWIZ using illumina® NovaSeq™/HiSeq® and the processing of the RNA-seq data was done using Bcl2fastq (v2.17.1.14). All samples passed quality control based on the results of FastQC. Raw reads (>21 million reads per sample) were aligned to the human reference genome (ENSEMBL, Homo_sapiens. GRCh37.91) via Hisat2 (v2.0.1). The transcript levels were displayed as reads per kilobase of transcript per million mapped reads (RPKM) obtained from HT-seq (V0.6.1), and the differential gene expression (DEG) was compared by RPKM profiles using Bioconductor package DESeq2 (V1.6.3) with a stringency level where the adjusted P value was equal to 0.05 (P adj < 0.05) and log2FC (Fold Change) was less than −1 or >1, P values for the DEGs were calculated using Wald test and P-adjusted values using the FDR/Benjamini–Hochberg approach. The Signaling pathways enrichment and Gene Set Enrichment Analysis (GSEA) was performed by using GSEA Java software (https://www.gsea-msigdb.org/gsea/index.jsp version 4.0) with MSigDb Hallmark gene sets. Enrichment scores (ES) and normalized enrichment scores (NES) were generated. Heat maps of DEGs were generated based on z-score values of normalized expression matrix from DESeq2 analysis in Gene-E (www.broadinstitute.org/GENE-E/).

Cells isolation and Flow Cytometry Analysis

In pristane-treated mice, peritoneal cells were harvested by lavage of the peritoneal cavity with 8 mL of PBS; kidney was minced and digested with the HBSS buffer containing 1 mg/ml collagenase type IV (Sigma C5138) at 37°C for 10 min as previous description. The tissue homogenates were passed through cell strainer (40 μm) and lysed with RBC lysis buffer to remove the remaining red blood cells. Cells were then re-suspended in 40% (w/v) Percoll-HBSS solution and overlaid onto 70% (w/v) Percoll-HBSS, followed by centrifuging at 2000 rpm for 30 min. The mononuclear cells (MNCs) were recovered from the 70 - 40% interphase and washed with HBSS to remove the residual Percoll. The isolated MNCs were re-suspended in FACS buffer and stained as follows. To determine the expression of IFN-α in inflammatory monocytes ($CD11b^{+}CD11c^{-}F4/80^{+}Ly6C^{hi}CCR2^{hi}$) and $CD11c^{+}$ dendritic cells ($1x10^{6}$/reaction) were incubated with a cocktail of surface marker antibodies (cocktail A) including anti-CD11b-BV650 (Cat.101239, Biolegend Inc.), anti-CD11c-BV421 (Cat. 562782, BD Bioscience Inc.), anti-F4/80-AF647 (Cat. MCA497RT, Bio-Rad.) anti-Ly6C-PE (Cat. 128008, Biolegend Inc.), and anti-CCR2-APC/Fire750 (Cat. 150630, Biolegend Inc.). The stained cells were then fixed and permeabilized with the CytoFix/perm solution (Cat. 554723 BD Bioscience Inc.), followed by washing with BD wash buffer and staining with anti-IFN-α-FITC (Cat. 22100-3, PBL Inc.) or matched isotype (anti-IgG1-

FITC (Cat. 400107, Biolegend Inc.) for 30 min on ice in the dark. After washing with FACS buffer, cells were suspended in 1xPBS and analyzed with the Beckman Coulter Cytoflex. To determine the myeloid immune population in the peritoneal cavity including inflammatory monocytes ($CD11b^{+}CD11c^{-}F4/80^{+}Ly6G^{-}Ly6C^{hi}CCR2^{hi}$), granulocytes ($CD11b^{+}CD11c^{-}F4/80^{+}Ly6C^{low}Ly6G^{+}$), macrophages ($CD11b^{+}CD11c^{-}F4/80^{hi}Ly6C^{-}Ly6G^{-}$), and dendritic cells ($CD11b^{+}CD11c^{+}$ $F4/80^{+}Ly6C^{-}Ly6G^{-}$), PEC ($1x10^{6}$/reaction) were incubated in the cocktail A with additional anti-Ly6G-FITC (Cat. 127605, Biolegend Inc.) in the cocktail. To determine the expression level of MHC II in the splenic dendritic cells, cells were incubated in the cocktail B including anti-CD11b-BV650, anti-CD11c-BV421, anti-F4/80-AF647, anti-Ly6C-PE, and anti-MHC II-BV510 (Cat. 107635, Biolegend Inc.). To determine the subpopulation of immune cells in the lymphoid lineage including T, B cells and plasma cells as well as the activated phenotype for these cells, splenocytes were incubated with a cocktail C of surface marker antibodies including anti-CD 3-APC-Cy7 (Cat. 553064, BD Bioscience Inc.), anti-CD 4-Alexa Fluor 647 (Cat. 553051, BD Bioscience Inc.), anti-CD19-BV510 (Cat. 115545, Biolegend Inc.), anti-CXCR5-AF488 (Cat. 142513, Biolegend Inc.), PD1-PE-Cy5.5 (Cat. 135207, Biolegend Inc) and anti-CD44-BV421 (Cat. 563970, BD Bioscience Inc.). Another cocktail D included anti-CD3-BV650 (Cat. 553064, BD Bioscience Inc.), anti-CD19-BV510 (Cat. 115545, Biolegend Inc.), anti-CD138-PE-Cy7 (Cat. 142513, Biolegend Inc.), anti-CD40-PE (Cat. 124609, Biolegend Inc.) and MHCII-FITC (Cat. 116405, Biolegend Inc). To determine the myeloid cells infiltrated in the kidney, MNC ($1x10^{6}$/reaction) from the kidney were incubated in the cocktail E including anti-CD11b-BV650, anti-CD11c-BV421, anti-Mac2-AF647 (Cat. 125419, Biolegend Inc.), anti-Ly6C-PE, anti-Ly6G-FITC, anti-CCR2-APC/Fire750 and anti-CX3CR1-PerCP5.5 (Cat.149009, Biolegend Inc.). To determine the DN T cells subset, splenocytes were incubated with a cocktail F of surface marker antibodies including anti-TCRβ-APC-Cy7 (Cat. 109219, Biolegend Inc.), anti-CD 4-Alexa Fluor 647 (Cat. 553051, BD Bioscience Inc.), anti-CD8-AF700 (Cat. 100729, Biolegend Inc.), anti-B220-AF488 (Cat. 103228, Biolegend Inc.), and anti-CD44-BV421 (Cat. 563970, BD Bioscience Inc.). All of the stained cells were washed with FACS buffer and resuspended in PBS and analyed with the Beckman Coulter CytoFlex. All of the data were processed using FlowJo software (version 10.8.1, Treestar).

Histopathologic assessment

Mouse kidneys were harvested in OCT (RA Lamb, U.K.) for cryosection or fixed in Formalin for paraffin-embedded 3 μm section. Periodic Acid-Schiff (PAS) stain or Hematoxylin and eosin (H&E) stain were performed. Each glomerulus was evaluated with severity (G score) as 0 (normal), 1 (endocapillary thickening or mesangial

proliferation <25% of glomerulus area), 2 (mesangial proliferation 25-50% of glomerulus area), 3 (mesangial proliferation between 50-75% of glomerulus area or fibrinoid necrosis), 4 (mesangial proliferation >75% area, crescent formation, or shrinkage of glomerulus), and 5 (total sclerosis or fibrosis) with 3 independent raters.

Immunofluorescence analysis

Frozen sections with 3-4 μm were fixed in acetone -20℃ for 7 minutes followed by blocking with 10% bovine serum albumin (BSA) for 1 hour in room temperature. Primary antibody of IgG (1:100, Cat. 115-545-166, Jackson immunoresearch) and FITC-conjugated C3 (1:100, Cat. GC3-90F-Z, In-Common Laboratories) were diluted in 3% BSA in 0.5% triton solution of phosphate-buffered saline (PBS) and applied on to the slides. Hoechst was counterstained. Images were detected with BX63 fluorescence microscope (CellSens software, Olympus).

Immunohistochemistry (IHC) staining and histology analysis

Paraffin-embedded kidney section was used for immunohistochemistry staining. Primary antibodies of IL-6 (AF-406, R&D), CD11b (Ab133357, Abcam), CD43 (553268, BD), fibrin (MABS2155, MilliporeSigma) and Ly6C (Ab15627, Abcam) were applied overnight at 4℃. The secondary antibodies including anti-Rat-HRP (Biolegend) and mouse or rabbit is Dako EnVision™ polymer HRP and were developed with Liquid DAB Chromogen System (Dako).

Reference

1. Chen ST, Chen L, Lin DS, Chen SY, Tsao YP, Guo H, et al. NLRP12 Regulates Anti-viral RIG-I Activation via Interaction with TRIM25. *Cell Host Microbe.* 2019;25(4):602-16.e7.

Supplementary Figure 1

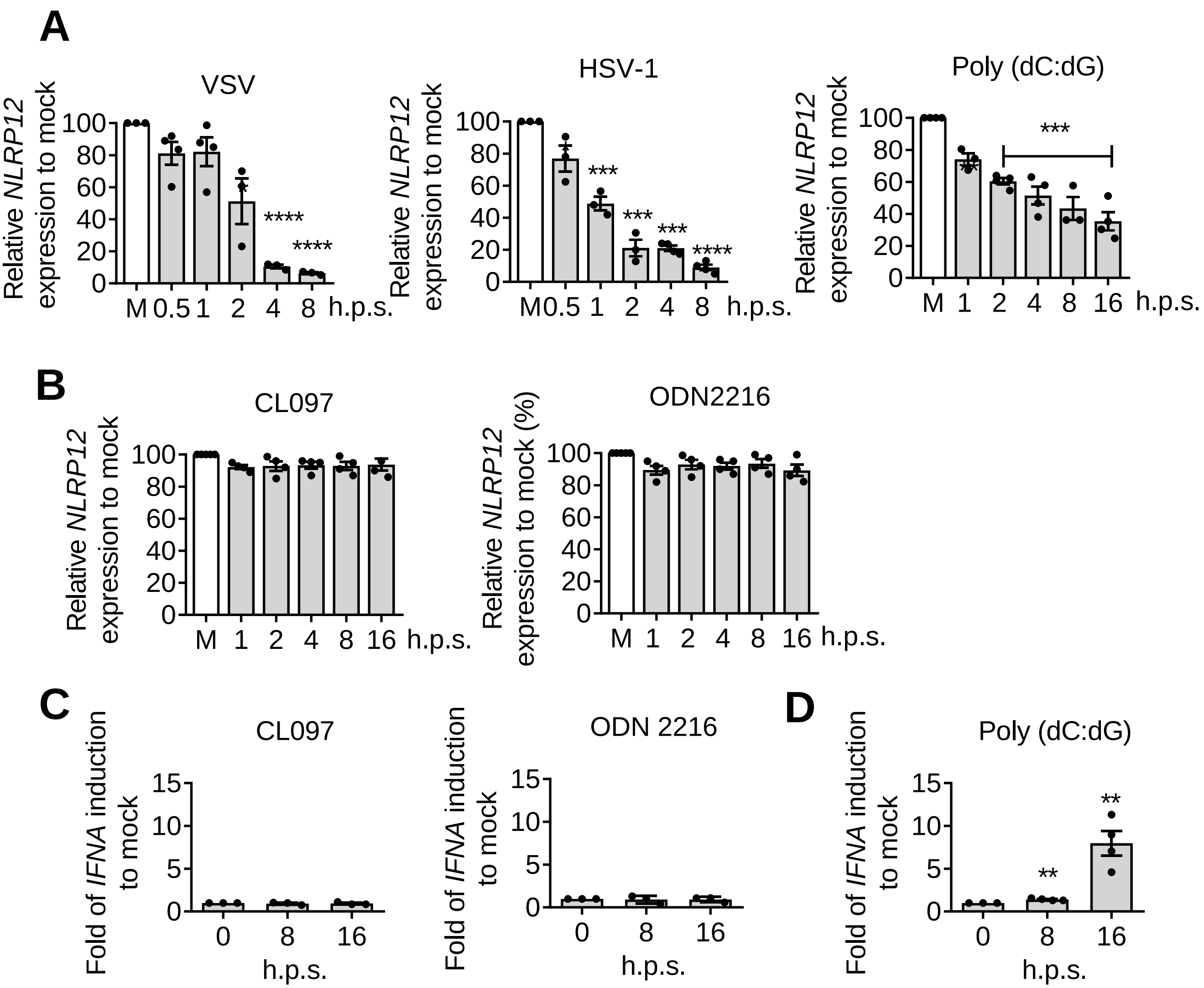

Supplementary Figure 1. Expression of *NLRP12* and *IFNA* transcripts after virus infection, nucleic acid transfection and ligand treatment. (**A**) Human monocytic THP-1 cells were infected with HSV-1 (m.o.i.=1), VSV (m.o.i.=1) or transfected with 5 µg/ml of poly (dC:dG), relative *NLRP12* mRNA expression was measured. (**B**) THP-1 cells were stimulated with 10 µg/ml of CL097 and ODN 2216, relative *NLRP12* mRNA or (**C**) *IFNA* mRNA expression was measured. (**D**) THP-1 cells were transfected with 5 µg/ml of poly (dC:dG), relative *IFNA* mRNA expression was measured. One-way ANOVA test (multiple samples to the mock control) was performed. Data represent as means ± SEM (n = 5), with *P < 0.05, **P < 0.01, and ***P < 0.001.

Supplementary Figure 2

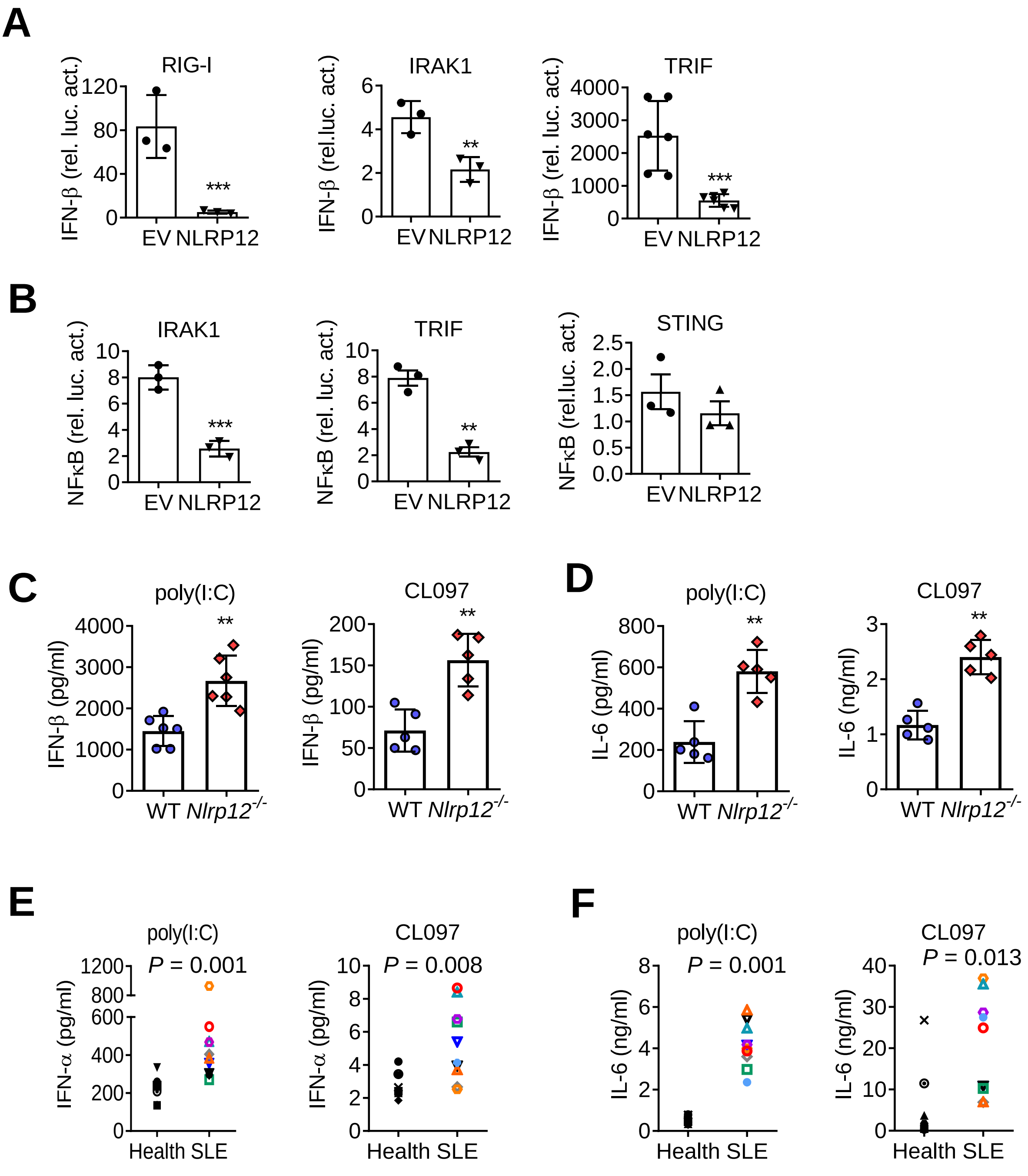

Supplementary Figure 2. NLRP12 involves in innate immune signaling to negatively regulate IFN-I and IL-6 production in response to nucleic acid stimulation. (**A** and **B**) HEK293 cells were transfected with 100 ng of (**A**) IFN-β or (**B**) NF-kB luciferase reporter with the internal control Renilla luciferase reporter pLR-TK plasmid and indicated plasmids (RIG-I, IRAK1, TRIF) in the presence of empty vector (EV, pCDNA3) or NLRP12-encoding plasmid (pCDNA3/HA-NLRP12, 300 ng/sample). Luciferase assays were performed 24 h post-transfection. (**C** and **D**) Mouse bone marrow derived dendritic cells from WT and *Nlrp12*$^{-/-}$ mice were transfected with poly (I:C) or treated with CL097 ligand at a concentrate of 5 μg/ml. Cytokine production was measured at 24 hours post stimulation by ELISA. (**E** and **F**) Human CD14^{+} primary monocytes from healthy donor (n = 8) and SLE patients (n = 10) were transfected with of 5 μg/ml of poly (I:C) or treated with CL097 ligand. Cytokine production was measured at 24 hours post stimulation by ELISA. For statistical analysis: (**A-D**) 2-tailed Student's t-test was performed. Data are presented as means ± SEM ($n \geq 5$). For (**E** and **F**) Mann-Whitney U test was conducted, with **P* < 0.05, ***P* < 0.01 and ****P* < 0.001.

Supplementary Figure 3

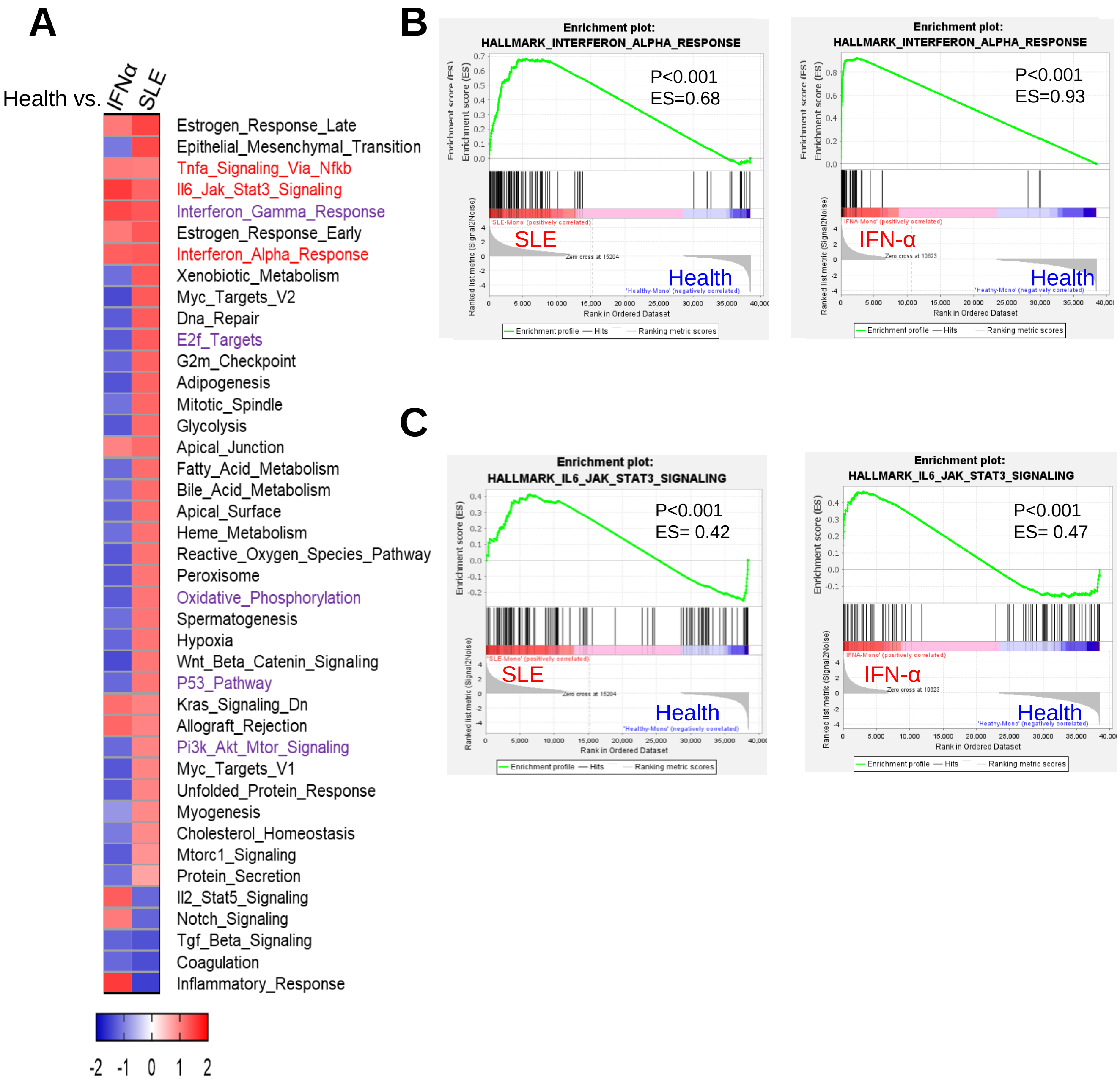

Supplementary Figure 3. GSEA analysis of RNA sequencing data in the IFN-α-treated monocytes and SLE monocytes. (**A**) The enriched differentially expressed genes (DEGs) in the biologically pathways were identified between IFN-α-treated and SLE monocytes from each of three samples. The DEGs are from two data sets: Healthy monocyte vs. IFN-α-treated healthy monocytes and Healthy monocyte vs. SLE monocyte. (**B** and **C**) Enrichment plots for two data sets enriched in GSEA MSigDb Hallmark analysis, showing the profile of the running enrichment score (ES) and positions of gene set members on the rank-ordered list.

Supplementary Figure 4

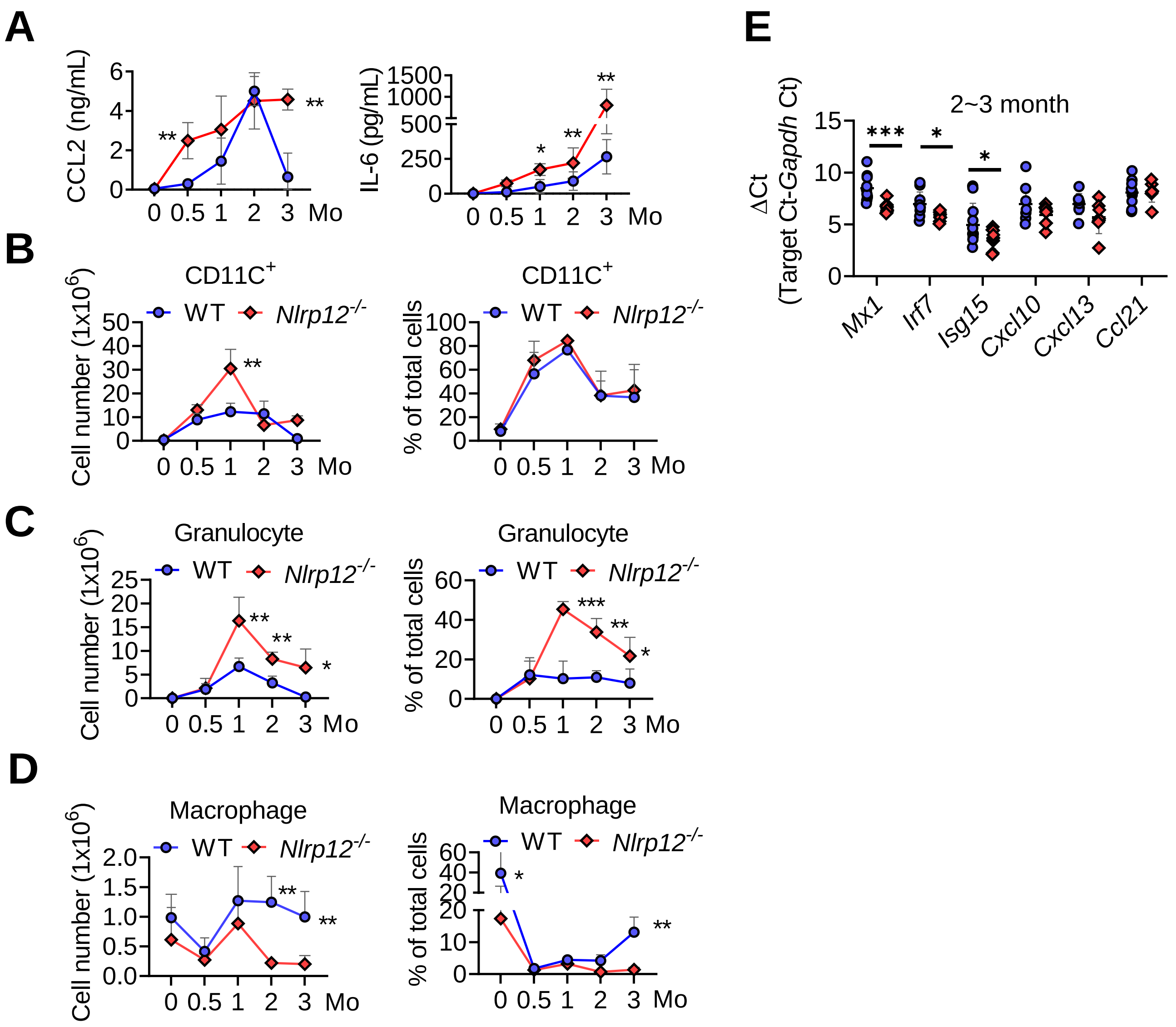

Supplementary Figure 4. NLRP12 deficient mice display greater immune cell infiltration and higher cytokine production in peritoneal cavity after pristane injection. Mice receiving one shot pristane injection were sacrificed at the indicated time points, and organs and tissues were harvested. (**A**) The production of CCL2 and IL-6 in the peritoneal lavage fluid was measured by CBA analysis. (Mo, month) (**B-D**) The immune phenotypes of the recruited peritoneal cells were characterized by multiple color FACS analysis and the number of distinct cell type were recorded. (**E**) The gene expression of the IFN signatures in peritoneal cells were measured; and data were displayed with delta Ct (△Ct), which stands for the absolute gene expression level of individual target. For statistical analysis: 2-tailed Student's t-test was performed. Data represent as means ± SEM ($n \geq 5$), with *$P < 0.05$, **$P < 0.01$ and ***$P < 0.001$.

Supplementary Figure 5

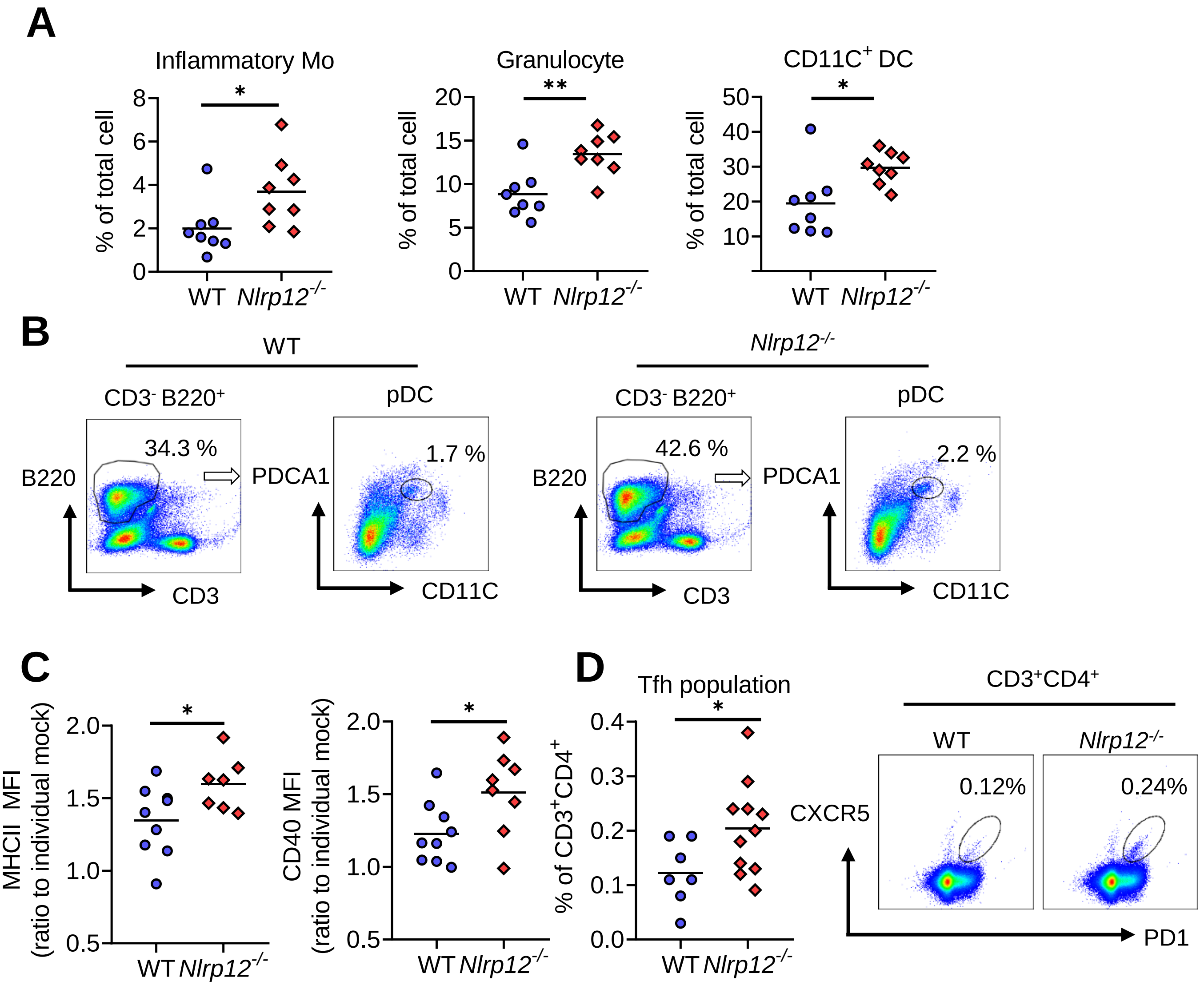

Supplementary Figure 5. Immune profiles of pristane treated- WT and NLRP12 deficient mice. Mice received one shot pristane injection were sacrificed and spleens were harvested to analyze immune cell populations at one-month post injection except additional mention. (**A**) Myeloid lineage of splenic cells was analyzed by multiple color FACS analysis at one-month post injection. (**B**) The frequency of the plasmacytoid dendritic cells in spleen were analyzed, representative dot blot was shown. (**C**) Relative surface expression of MHC class II and CD40 expression by $CD19^+$ cells to the mock control was analyzed by FACS analysis. **(D**) The frequency of the splenic follicular T helper cells in $CD3^+CD4^+$ population was analyzed at the 3rd month post pristane injection. For statistical analysis: (**A, C, D**) 2-tailed Student's t-test was performed. Data represent as means ± SEM ($n \geq 7$), with $*P < 0.05$ and $**P < 0.01$.

Supplementary Figure 6

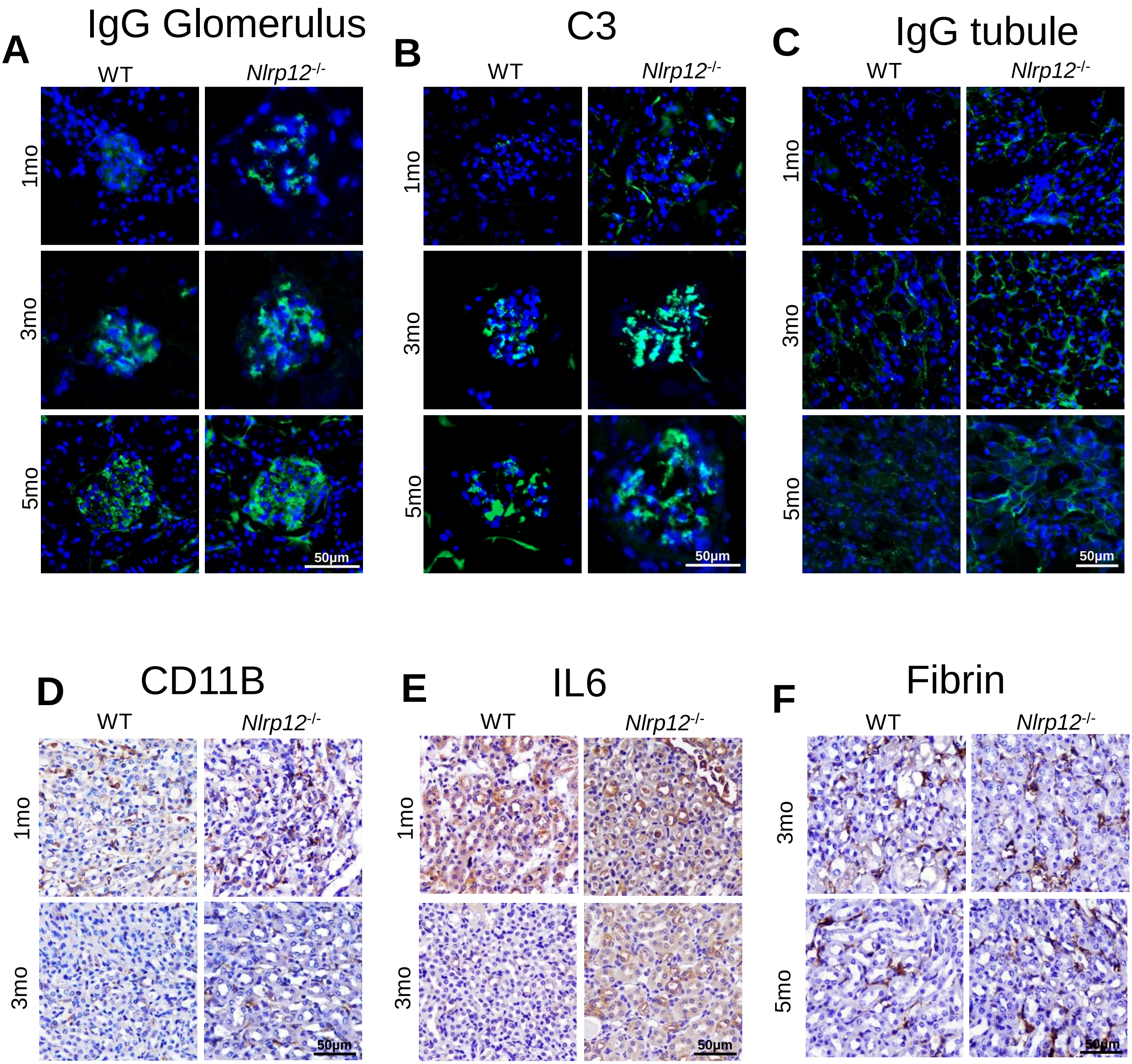

Supplementary Figure 6. Immunofluorescence and immunohistochemical analysis of kidney sections. Representative images of (**A**) IgG deposition and (**B**) C3 deposition in glomerulus at 1st, 3rd, 5th months of pristane-treated WT and *Nlrp12*$^{-/-}$ mice. (**C**) Representative images of IgG deposition in renal tubule of pristane-treated mice. Representative images of IHC staining for (**D**) CD11B^{+}, (**E**) IL-6 and (**F**) fibrin in renal tubules of pristane-treated mice. Scale bar, 50 μm.

Supplementary Figure 7

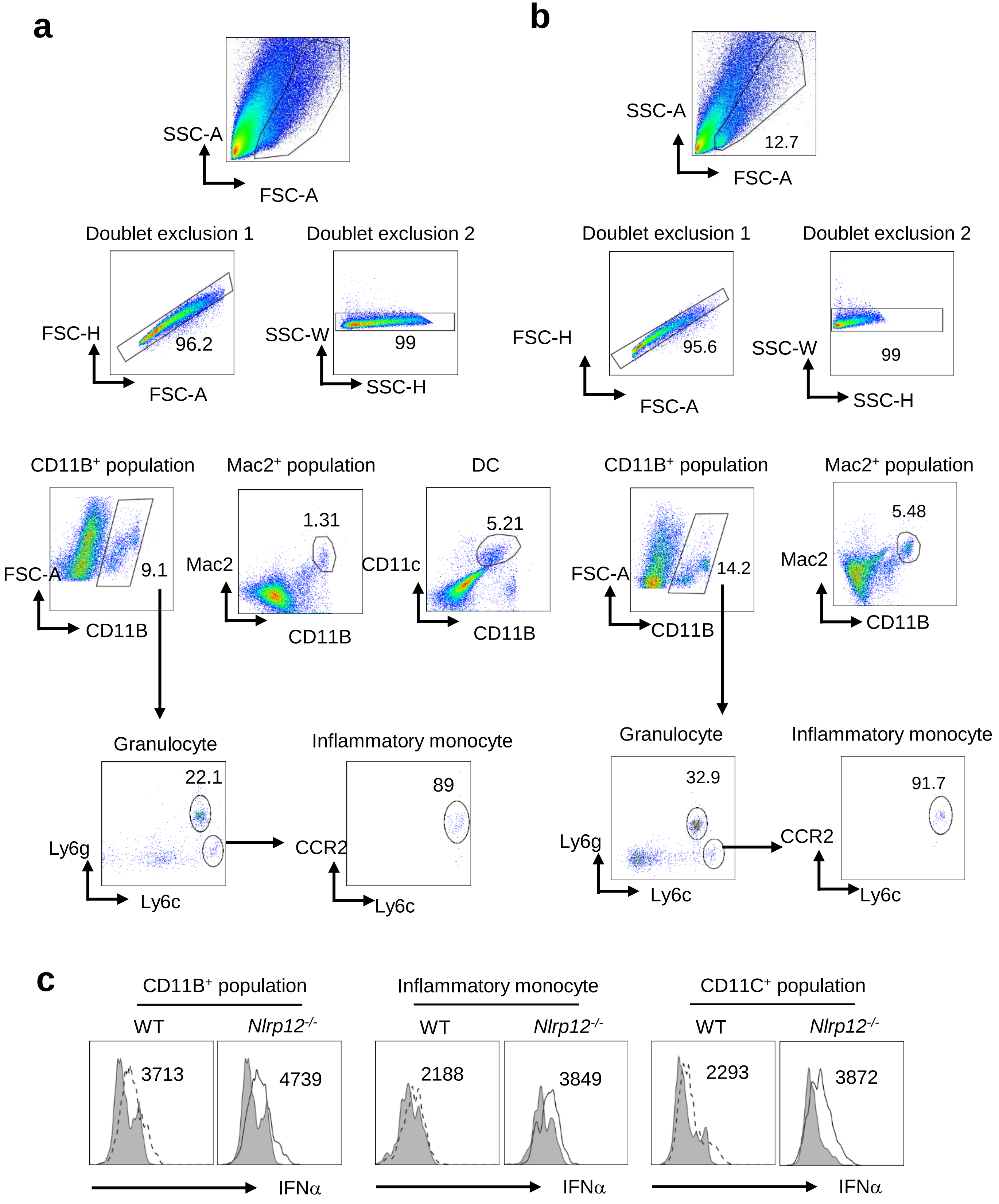

Supplementary Figure 7. Flow cytometry gating schemes. (**A** and **B**) Gating scheme for the analysis of myeloid lineage cell population in the kidney including $CD11B^+$ cells, $Mac2^+$ macrophage, $CD11C^+$ DC, inflammatory monocyte and granulocyte from WT mice at 1st and 3rd month post pristane-challenge respectively. (**C**) Representative histogram showing IFN-α expression in each population of myeloid cells isolated from the kidney of the pristane-treated WT and *Nlrp12*$^{-/-}$ mice. Numeric indicates mean fluorescence intensity (MFI) of positive IFN-α staining. Shaded areas represent isotype controls.

Supplementary Figure 8

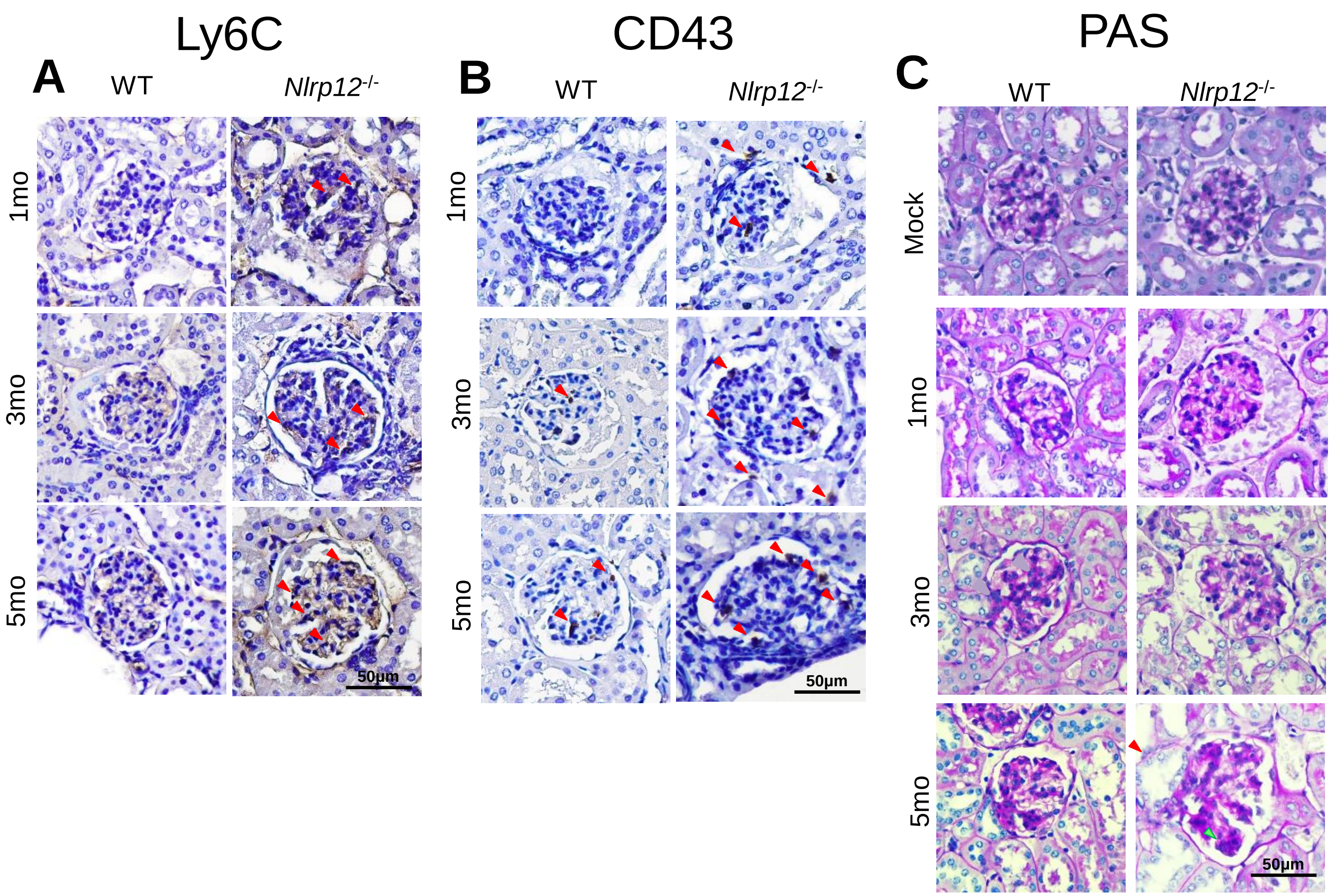

Supplementary Figure 8. Cyto-histologic change of glomerulus in pristane treated mice. Immune cells were stained with IHC from WT and *Nlrp12*$^{-/-}$ mice for Representative images of IHC staining for (**A**) Ly6C and (**B**) CD43 in glomerulus. (**C**) Representative images of PAS staining for glomerulus.

Supplementary Figure 9

Equipment:
Universal Imaging MetaMorph® Imaging System, Molecular Devices

Mesangial Area Quantification:

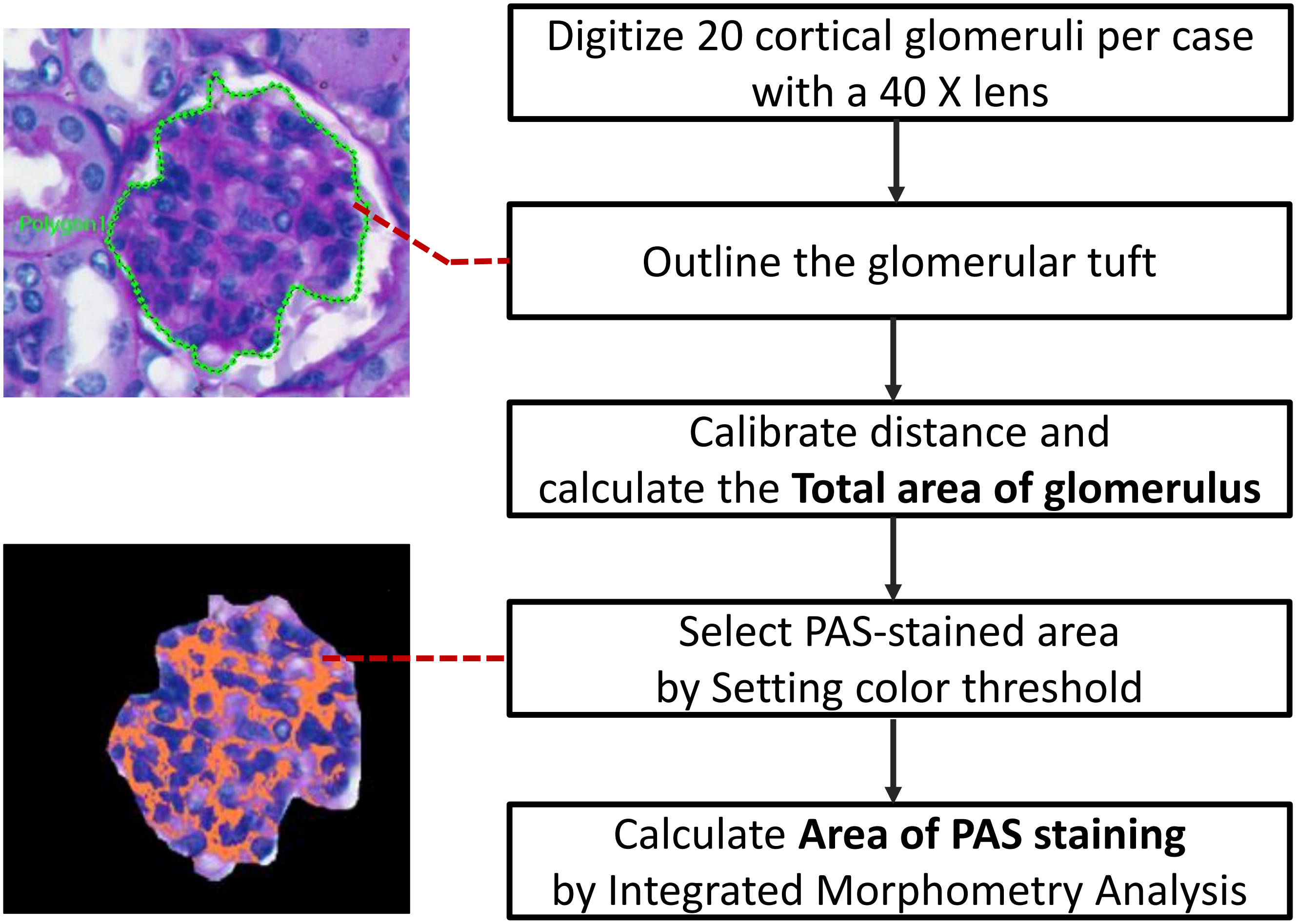

Ref: AMDCC (Animal models of Diabetic-Complications Consortium) protocols- Mesangial Index Quantification

Supplementary Figure 9. Flowchart of calculating PAS staining area of kidney section by metamorph® software.

Supplementary Figure 10

A

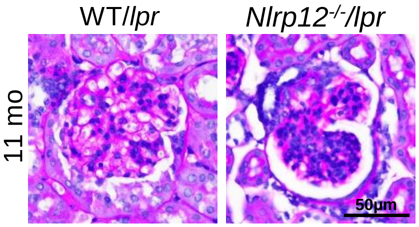

B

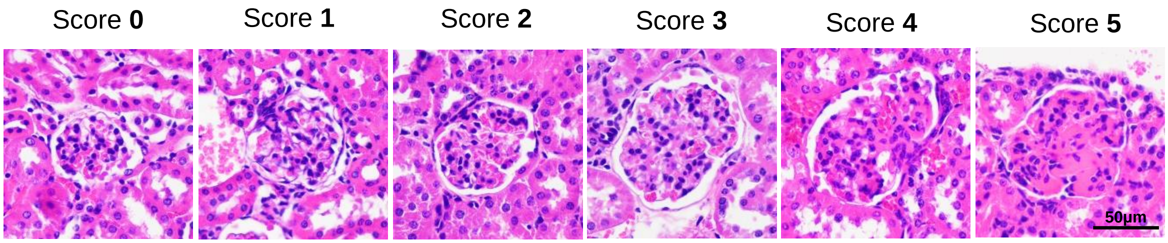

Supplementary Figure 10. PAS stain and representative H&E stain of glomerulus. (**A**) PAS stain of WT/lpr and *Nlrp12*$^{-/-}$/lpr mice in 11 month. (**B**) Representative G score from 0-5 of H&E stain. Scale bar, 50 μm.

Supplementary Figure 11

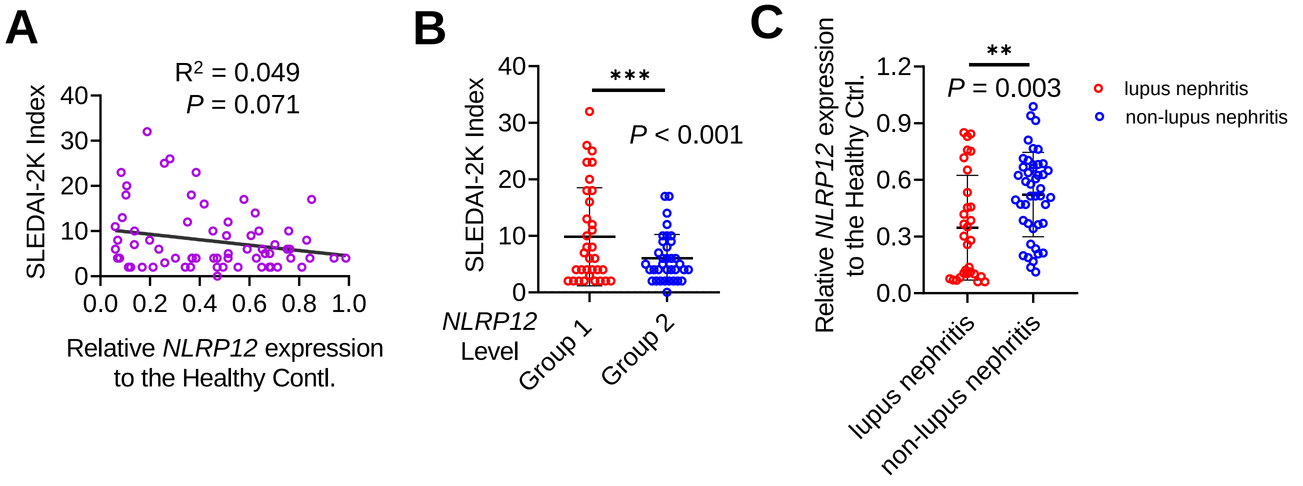

Supplementary Figure 11. Analysis of clinical parameters with *NLRP12* expression. (**A**) Linear regression of SLEDAI and relative *NLRP12* expression. (**B**) SLEDAI under grouping with relative *NLRP12* expression below 0.43 (Group 1) and above 0.43 (Group 2). (**C**) Relative *NLRP12* expression in lupus nephritis and non-lupus nephritis PBMCs to the healthy control (Ctrl.) was determined by RT-QPCR. For statistical analysis: (**A**) Spearman's correlation was conducted; (**B** and **C**) 2-tailed Student's t test. Data represent mean ± SEM, with $^{**}P < 0.01$ and $^{***}P < 0.001$.

Supplementary Figure 12

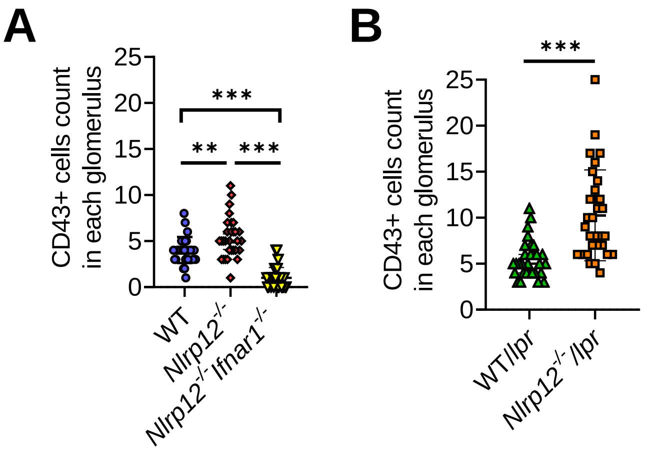

Supplementary Figure 12. CD43 positive cells count of each glomerulus at 9th month in different animal model. Total 30 glomerulus of different animal model was evaluated with CD43 positive cells in each glomerulus at 9th month. (**A**) Pristane induced lupus like model under WT, *Nlrp12$^{-/-}$*, and *Nlrp12$^{-/-}$Infar$^{-/-}$* mice. Less CD43 cells were observed in *Nlrp12$^{-/-}$Infar$^{-/-}$* mice. (**B**) Lupus prone mice with WT/lpr and *Nlrp12$^{-/-}$*/lpr mice. For statistical analysis: (**A**) One-way ANOVA was performed; (**B**) Mann-Whitney U Test was performed. Data represent mean ± SEM, with $^{**}P < 0.01$ and $^{***}P < 0.001$.

Supplementary Figure 13

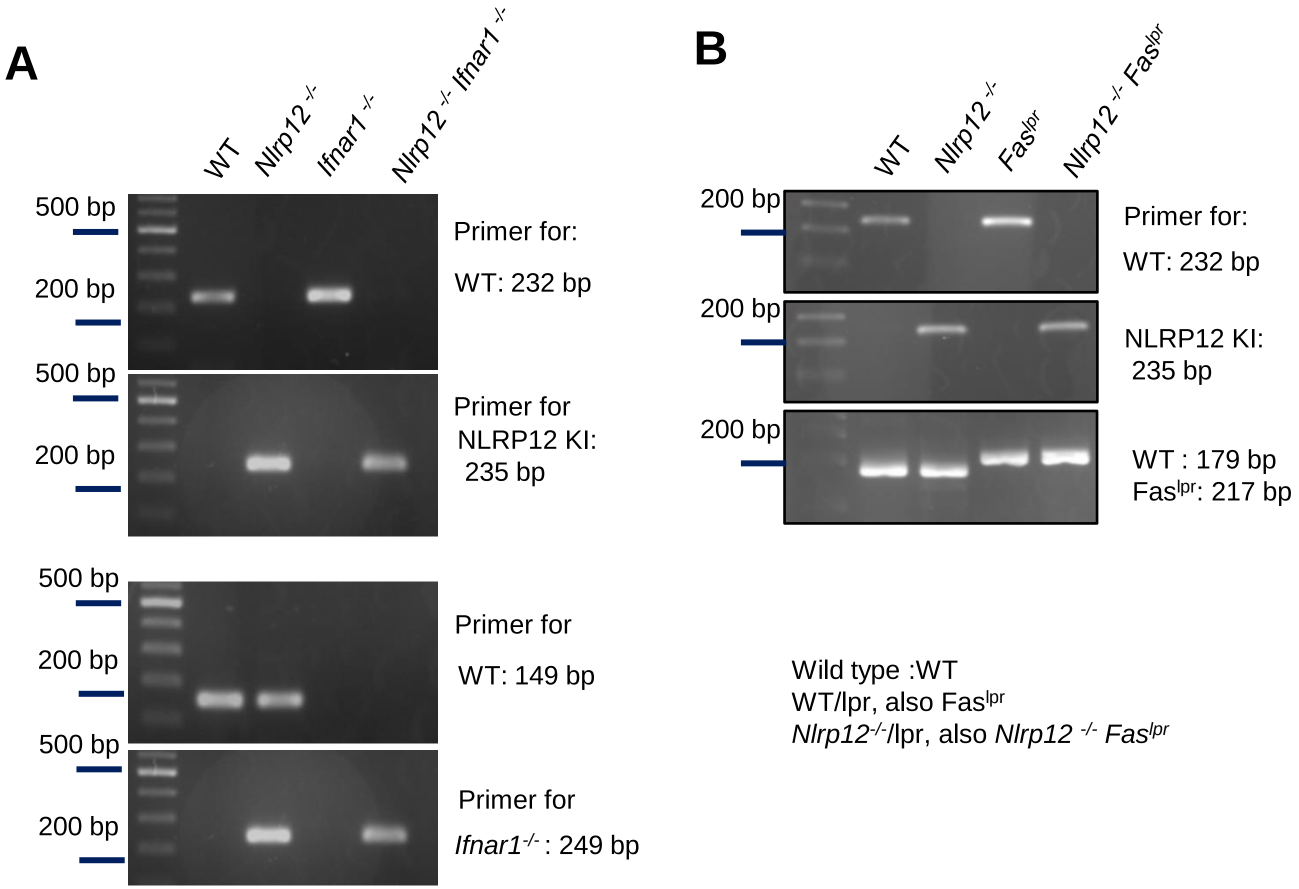

Supplementary Figure 13. Generation of NLRP12 and IFNAR1 double knockout mice, and NLRP12 deficient lupus prone mice. Genotyping was conducted by PCR using corresponding primer sets.

Table S1. Characteristics of SLE patients

Chart#	Relative *NLRP12* expression	Relative *IFNA* expression	Age	Sex	WBC	Hgb (g/dl)	Platelet (*1000)	C3	C4	ANA	dsDNA Ab	Lupus nephritis	SLEDAI-2K
**1**	**.46993**	**1.24573**	55	F	4500	13	120	73	14	320	24	0	2
**2**	**.21237**	**1.61948**	54	M	8400	12	219	69	18	80	68	0	2
**3**	**.62336**	**1.26038**	49	F	2300	12	117	32	2	320	74	0	14
**4**	**.08830**	**2.92163**	61	F	4800	12	198	139	36	640	1	1	13
**5**	**.25728**	**3.81370**	67	F	5900	9	85	54	9	N.D.	16	1	25
**6**	**.11300**	**1.54900**	36	F	10500	10	406	78	17	320	17	1	2
**7**	**.76227**	**1.17213**	49	F	14900	13	419	105	18	640	134	0	6
**8**	**.68000**	**3.41000**	29	F	5500	12	283	114	26	160	19	0	2
**9**	**.62812**	**.64834**	50	F	4800	11	234	80	22	320	N.D.	0	4
**10**	**.81100**	**.77500**	39	F	8200	13	436	59	10	320	1	0	2
**11**	**.36625**	**1.40101**	41	F	3100	5	99	*	*	320	22	1	18
**12**	**.16792**	**2.48288**	46	F	3900	10	176	89	17	160	155	0	2
**13**	**.59095**	**.19423**	50	F	2900	12	41	76	21	320	21	0	6
**14**	**.10264**	**.79645**	38	F	3300	11	181	*	*	640	11	1	18
**15**	**.20805**	**2.74691**	59	F	8400	14	211	137	26	320	213	0	2
**16**	**.45301**	**4.18904**	40	F	4600	13.8	273	49.6	20.3	320	1.8	1	10
**17**	0.50756	3.55384	57	F	3800	12	176	46	5	1280	72	0	9

Normal range of dsDNA Ab: <10 IU/ml

* Data Missing
N.D. Non detectable

Table S1. Characteristics of SLE patients

Chart#	Relative *NLRP12* expression	Relative *IFNA* expression	Age	Sex	WBC	Hgb (g/dl)	Platelet (*1000)	C3	C4	ANA	dsDNA Ab	Lupus nephritis	SLEDAI-2K
**18**	0.351	4.258	32	F	7200	11	309	42	6	160	N.D.	1	12
**19**	0.385	3.606	31	F	2300	12.2	233	48.8	11.2	640	341	1	23
**20**	0.06057	6.40759	39	M	6100	14	289	112	22	160	123	1	6
**21**	0.07	6.14	25	F	10000	13	288	75	7	320	105	1	8
**22**	0.25938	1.32099	60	F	2800	10	146	97	21	160	35	0	3
**23**	0.0687	2.76	33	F	9900	9	336	101	12	N.D.	1	1	4
**24**	0.11261	9.67201	37	F	6900	12	137	*	*	1280	N.D.	0	2
**25**	0.66288	3.31143	25	F	6900	11.9	248	135.2	19	1280	4.4	0	5
**26**	0.27965	2.18788	83	M	13400	9.7	92	57.6	16.7	2560	444	1	26
**27**	0.1375	5.19	34	F	7200	13	252	91	20	160	80	1	10
**28**	0.08322	9.35853	30	F	3300	10	229	63	21	1280	154	1	23
**29**	0.68199	0.53823	38	F	3500	13	198	75	8	160	89	0	5
**30**	0.514	2.605	39	F	3000	12	133	70	13	640	52	0	12
**31**	0.47	6.66	28	F	5500	11	234	73	16	N.D.	27	0	4
**32**	0.106	1.13	22	F	6500	10	97	22	2	5120	88	1	20
**33**	0.06	6.02	47	F	11300	9	161	118	22	80	2	1	11
**34**	0.57737	2.2097	18	F	7700	12.3	332	55.9	5.5	640	68	0	17

Normal range of dsDNA Ab: <10 IU/ml

*   Data Missing
N.D. Non detectable

Table S1. Characteristics of SLE patients

Chart#	Relative *NLRP12* expression	Relative *IFNA* expression	Age	Sex	WBC	Hgb (g/dl)	Platelet (*1000)	C3	C4	ANA	dsDNA Ab	Lupus nephritis	SLEDAI-2K
**35**	0.34126	4.9948	37	F	4800	12.1	254	94.4	14.6	640	1	0	2
**36**	0.63805	2.3741	26	F	4055	11.3	236	83.1	14.1	5120	60	0	10
**37**	0.68623	1.361	23	F	8355	11.4	137	105.5	30.1	160	29	0	2
**38**	0.64956	5.7879	50	F	6500	15.8	232	87.2	17.2	N.D.	2.8	0	2
**39**	0.3855	3.37956	51	F	3000	14.5	50	82.6	10.1	160	17	0	4
**40**	0.47101	3.9722	24	F	4100	13.8	204	108.3	22	80	1.2	0	0
**41**	0.36965	6.8258	19	F	6500	13.3	287	73.5	9.7	N.D.	30	0	4
**42**	0.23511	4.7289	47	F	5600	13.7	292	110.1	19.2	2560	18	0	6
**43**	0.13651	5.47963	20	F	4350	11.6	313	81.1	23.3	160	2.1	0	7
**44**	0.60595	2.09605	58	M	5200	14.2	196	88.3	14.3	640	28	0	9
**45**	0.19824	9.77266	42	F	2600	12.4	179	77.7	9.9	5120	178	1	8
**46**	0.70275	3.62591	39	F	5900	11.2	180	78.8	23.4	1280	9.8	0	7
**47**	0.36861	9.7736	37	F	4500	10.5	290	49.4	4.6	160	208	0	4
**48**	0.84343	1.7089	59	F	5000	10.3	164	90.7	32.2	160	0.5	1	4
**49**	0.652	0.455	45	F	8440	10.3	317	77.5	37.2	80	5.2	1	6
**50**	0.363	8.444	49	F	6390	14.3	217	90.7	17	160	71	0	2
**51**	0.83	6.248	37	F	6520	13.6	263	63.1	5.3	160	79	1	8

Normal range of dsDNA Ab: <10 IU/ml

* Data Missing
N.D. Non detectable

Table S1. Characteristics of SLE patients

Chart#	Relative *NLRP12* expression	Relative *IFNA* expression	Age	Sex	WBC	Hgb (g/dl)	Platelet (*1000)	C3	C4	ANA	dsDNA Ab	Lupus nephritis	SLEDAI-2K
**52**	0.94	2.813	40	F	6620	11.7	262	66.4	23.6	80	41	0	4
**53**	0.988	0.536	33	F	7460	9.7	253	66.4	5.4	160	101	0	4
**54**	0.302	17.845	54	M	7810	12.3	200	86.8	13.3	N.D.	3.3	1	4
**55**	0.766	4.946	60	F	6120	12.9	234	95.4	14.1	320	32	0	4
**56**	0.758	2.037	20	F	10710	10.9	392	57	6.5	320	84	1	10
**57**	0.456	3.012	28	F	4180	12.4	219	66.7	6.2	160	110	1	4
**58**	0.514	1.529	42	F	6060	13.2	269	78.4	12.2	640	62	0	4
**59**	0.554	4.807	40	F	9560	14.5	332	133.7	30.2	160	21	0	2
**60**	0.713	7.565	44	F	6310	12.3	308	127.7	37.5	N.D.	21	0	2
**61**	0.418	8.499	26	F	7610	9.8	241	73.2	11.1	160	147	1	16
**62**	0.85	4.938	38	F	4720	7.2	227	56.2	10.5	80	18	1	17
**63**	0.515	2.934	42	M	7680	16.7	125	74	17.6	80	26	0	5
**64**	0.188	10.698	20	F	2800	9.8	134	42.6	3.9	640	202	0	32
**65**	0.494	3.121	45	F	4100	13	292	91.1	17.2	160	40	0	2
**66**	0.077	7.446	53	F	4300	12.4	191	77.7	15.6	1280	29	1	4
**67**	0.122	10.032	60	F	4600	10.8	119	70.3	19.9	320	1.3	1	2
**68**	0.752	10.663	28	F	5830	10.5	210	33.7	3	640	131	1	6

Normal range of dsDNA Ab: <10 IU/ml

*   Data Missing
N.D. Non detectable

Table S2. Characteristics of SLE patients

Chart#	Serum IFN-α (pg/mL)	THP1 *NLRP12* expression	WBC	C3	CRP	dsDNA Ab	SLEDAI-2K
**1**	4.59859	0.61565	2300	48.8	0.05	341	25
**2**	351.97631	0.31728	8400	69.5	0.15	51	26
**3**	537.30082	0.83304	3300	55.2	0.08	36	12
**4**	511.52163	0.67135	1700	25.02	0.89	1.9	19
**5**	23.20395	0.60527	5800	67.1	0.03	1.5	10
**6**	260.08692	0.27818	3000	82.6	2.71	17	20
**7**	39.39308	0.37927	4980	76.8	0.04	1.5	12
**8**	275.83955	0.14198	2800	42.6	4.65	202	32
**9**	61.16028	0.17283	4720	56.2	0.21	18	17
**10**	35.03514	0.62770	2300	59.9	2.23	35	24
**11**	39.11083	0.33422	3710	36.8	0.09	450	51
**12**	6.44886	0.58831	8100	58.5	1.47	4.4	34

Normal range of dsDNA Ab: <10 IU/ml

Table S2. Characteristics of SLE patients

Chart#	Serum IFN-α (pg/mL)	THP1 *NLRP12* expression	WBC	C3	CRP	dsDNA Ab	SLEDAI-2K
**13**	undetectable	1.45475	6100	74.1	0.05	33	4
**14**	undetectable	0.47754	4900	82.6	0.05	60	16
**15**	undetectable	0.58618	6500	87.2	0.34	2.8	4
**16**	undetectable	1.02171	6600	59.6	0.03	1.7	6
**17**	undetectable	1.53194	4000	*	0.03	12	3
**18**	undetectable	0.66400	5700	88.3	0.57	50	4
**19**	undetectable	0.95635	2500	70.9	0.25	86	7
**20**	undetectable	1.43723	6400	64.7	0.1	1.4	14
**21**	undetectable	1.26287	4800	83.8	0.03	37	6
**22**	undetectable	0.95635	7400	74.8	4.35	59	25
**23**	undetectable	0.88471	7680	74.0	0.18	26	5
**24**	undetectable	1.36811	7810	86.8	0.10	3.3	4

Normal range of dsDNA Ab: <10 IU/ml

Table S3. Characteristics of SLE patients

Chart#	Age	Sex	Past Clinical Manifestations From Chart Review				WBC	Hgb (g/dl)	Platelet (*1000)	C3	C4	ANA	dsDNA Ab	SLEDAI-2K
			Skin	Nephritis	Neurologic signs	DVT/Abortions/ CV events/Stroke								
**1**	34	F	+	+	+	+	3400	12	232	46.3	11.3	640	343	4
**2**	85	M	+	+	+	+	7400	9.9	269	74.8	36.2	640	59	4
**3**	57	M	+			+	5700	13.3	234	67.3	15.4	640	50	6
**4**	33	F	+		+		8400	11.6	235	69.5	14.4	640	97	4
**5**	20	F	+				4800	13.1	305	83.8	18.3	160	37	6
**6**	44	F	+	+	+	+	3300	8.4	62	55.2	23.6	160	36	5
**7**	47	F			+	+	4000	10.8	32	78.6	22.1	640	12	5
**8**	40	F			+		5500	13.4	377	65.8	15.9	320	1.7	2
**9**	48	F			+	+	6400	10.3	314	64.7	12.7	160	1.4	2
**10**	37	F	+			+	9900	13.8	301	103.6	19.9	160	1.4	2

Normal range of dsDNA Ab: <10 IU/ml

DVT deep vein thrombosis
CV cardiovascular

Table S4. Human and mouse Q-PCR primers used in this study

Gene	Forward	Reversed
NLRP12	cag act cca gaa act gtg g	gcg ttg ttg gtc agg taa g
IFNA	acc tgg aag cct gta tga tac	gat ttc tgc tct gac aac ctc
RUNX1	gaa ttc cat gcg tat ccc cg	aga tct tca gta ggg cct cca cac
GAPDH	cca tca ctg cca ccc aga aga c	ggc agg ttt ttc tag acg gca g
Mx1	gat ccg act tca ctt cca gat gg	cat ctc agt ggt agt caa ccc
Irf7	gga cct ctt gct tca ggt tct	agg gtt cct cgt aaa cac gg
Isg15	cag aag cag act cct taa ttc	aga cct cat ata tgt tgc tgt g
Cxcl10	aag cta tgt gga ggt gcg ac	cta ggg agg aca agg agg gt
Cxcl13	tct ctc cag gcc acg gta ttc t	acc att tgg cac gag gat tca c
Ccl21	tga gct atg tgc aaa ccc tga gga	tga ggg ctg tgt ctg ttc agt tct
Nlrp12	cct ctt tga gcc aga cga ag	gcc cag tcc aac atc act tt
Gapdh	gga gga acc tgc caa gta tg	tgg gag ttg ctg ttg aag
